# Transcriptional Dynamics of Receptor-Based Genes Reveal Immunity Hubs in Rice Response to *Magnaporthe oryzae* Infection

**DOI:** 10.3390/ijms26104618

**Published:** 2025-05-12

**Authors:** Fatma Salem, Ahmed ElGamal, Xiaoya Tang, Jianyuan Yang, Weiwen Kong

**Affiliations:** 1 College of Plant Protection, Yangzhou University, Yangzhou 225009, China; fss00@fayoum.edu.eg (F.S.); xiaoya__tang@163.com (X.T.); 2 Faculty of Agriculture, Fayoum University, Fayoum 63514, Egypt; 3 Virus and Phytoplasma Research Department, Plant Pathology Research Institute, Agricultural Research Center, Giza 12619, Egypt; ahmed.elgamal@arc.sci.eg; 4 Plant Protection Research Institute, Guangdong Academy of Agricultural Sciences, Guangzhou 510640, China

**Keywords:** rice blast, extracellular receptors, PTI, *Magnaporthe oryzae*

## Abstract

Rice blast caused by *Magnaporthe oryzae* (MOR) reigns as the top-most devastating disease affecting global rice production. Pattern-triggered immunity (PTI) is crucial for mitigating plant responses to pathogens. However, the transcriptional dynamics of PTI-related genes in rice response to MOR infection remain largely unexplored. In this study, we performed a meta-analysis of 201 RNA sequencing and 217 microarray datasets to investigate the transcriptional dynamics of rice under MOR infection at various infection stages. The transcriptional dynamics of extracellular/cytoplasmic receptor kinase genes (*RLKs*, *RLCKs*, *WAKs*) and downstream signaling intermediates, including mitogen-activated protein kinases (MAPKs) and Ca^2+^-related signaling genes, were identified as immunity hubs for PTI. Extracellular/cytoplasmic receptors were predominantly induced, in contrast to a marked decrease in the repression of these genes. Notably, a maximum of 141 and 154 receptor-based genes were frequently induced from the microarray and RNA-seq datasets, respectively. Moreover, 31 genes were consistently induced across all the transcriptomic profiles, highlighting their pivotal role in PTI-activating immunity regulation in rice under MOR stress. Furthermore, protein–protein interaction (PPI) analysis revealed that cytoplasmic receptor-based genes (RLCKs) and MAPK(K)s were highly interconnected. Among them, four core *MAPKK* genes, including *SMG1*, *MKK1*, *MKK6*, and *MPKK10.2*, were identified as the most frequently interconnected with receptor-based genes or other MAPKs under MOR infection, suggesting their critical role as intermediates during downstream signaling networks in response to MOR infection. Together, our comprehensive analysis provides insights into the transcriptional dynamics of receptor-based genes and downstream signaling intermediates as core PTI-related genes that can play crucial roles in modulating rice immune responses to MOR infection.

## 1. Introduction

Rice (*Oryza sativa*) is an important crop for global food security and serves as a model monocotyledonous plant for studying host defense mechanisms against pathogens. *Magnaporthe oryzae* (MOR), a fungal pathogen, is a major threat to rice cultivation and causes substantial yield losses in rice production worldwide [1,2]. MOR pathogen ranks among the top significant and destructive fungal pathogens affecting rice, simultaneously causing blast diseases on more than 50 plant species [3]. Plants have evolved a sophisticated immune system to oppose infection by various pathogens through a dual, two-tiered immune system [4]. The first layer, known as PAMP-triggered immunity (PTI), is initially activated upon the recognition of conserved microbial patterns known as pathogen-associated molecular patterns (PAMPs) by pattern recognition receptors (PRRs) on the cell surface [5]. Additionally, plants have developed a second defense layer activated by the recognition of effectors through an intracellular nucleotide-binding domain and leucine-rich repeat (NB-LRR) immune receptors (NLRs) [6]. This immune system is called effector-triggered immunity (ETI). ETI often induces a hypersensitive response (HR) that is accompanied by cell death, which contributes to the restriction of pathogen proliferation. However, PTI machinery mostly contributes to the early signal downstream defense response. This process is driven by a rapid influx of cytosolic calcium (Ca^2+^), initiating signal transduction and triggering MAPK cascades [7,8,9]. Both PTI and ETI are involved in the downstream activation of robust plant defense mechanisms against pathogen infections, including the biosynthesis of salicylic acid (SA) and defense phytohormones that mediate systemic acquired resistance (SAR) [10,11]. However, recent studies revealed extensive synergy between PTI and ETI, leading to mutual potentiation and interdependency, which is critical for a full immune response [12]. Despite differences in their triggers and dynamics, they share several overlapping processes, including the production of reactive oxygen species (ROS), transcriptional reprogramming of pathogenesis-related (PR) proteins, and the accumulation of secondary metabolites [11].

At the forefront of the PTI defense mechanism are receptor-based genes, which encode proteins responsible for detecting PAMPs [13]. PTI is a mechanism mediated by PRRs, such as receptor-like kinases (RLKs), which are anchored in the plasma membrane [14,15,16,17]. This innate immune system includes receptors that actively recognize all pathogen classes and induce defense signaling responses that culminate in the expression of host resistance [17]. Surface-localized plant immune receptors encoding RLKs or receptor-like proteins (RLPs) play an important role in plant innate immunity. The RLK family and the associated receptor-like cytoplasmic kinases (RLCKs) have expanded in plants because of selective pressure from environmental stress and evolving pathogens. RLKs are mainly involved in recognizing a wide range of pathogen-associated molecules, such as cell wall fragments and extracellular alterations triggered by pathogen invasion [16,18]. Likewise, RLCKs are a cytoplasmic subclass of RLKs and can be crucial in downstream signal transduction, linking pathogen perception to the activation of coping mechanisms [19,20]. RLCKs lack an extracellular domain and tend to interact with RLKs and other membrane-localized proteins to participate in signal relay [20]. The plant cell wall acts as a structural defense against pathogen invasion and a hub for receptors that identify microbial effectors, conserved pathogen-associated molecular patterns, and damage from infections. One notable group of defense-related kinases is the wall-associated kinase (WAK) family. These kinases belong to the receptor-like kinase (RLK) class and are distinguished by their serine/threonine kinase domain and an epidermal growth factor-like (EGF-like) domain [18,21]. The extracellular domains of WAKs exhibit a strong ability to bind small pectic oligosaccharides, which are released during the pathogen-induced cell wall degradation [22]. Following receptor activation, downstream signaling pathways, including MAPK phosphorylation and calcium ion (Ca^2+^) sensors, are triggered to mediate transcriptional reprogramming [13,23,24]. These pathways involve calcium ion influx, activation of MAPKs, production of ROS in the extracellular space, transcriptional reprogramming, and the fortification of cell walls to enhance physical defenses [25].

Understanding the intricate relationships between hosts and pathogens relies heavily on deciphering transcriptional dynamics and genome reprogramming [26]. In addition, some studies have provided paradigms about RLK/RLCKs functions, but a lack of understanding of crop RLK/RLCKs regarding their role in host–pathogen interactions undermines their application.

Some resistance genes have been identified in response to MOR infection in rice, while little is known about the transcript dynamics of genes involved in PTI and signaling intermediates [27,28,29]. In addition, the role of several receptor-based genes, including extracellular and intracellular receptors in rice immunity against MOR, remains elusive. This study presents a comprehensive meta-analysis of transcriptomic datasets, leveraging a large collection of RNA-seq and microarray profiles. Our study aimed to elucidate the transcriptional features of receptor-based genes alongside other downstream signaling intermediates after MOR infection, uncovering key regulators of rice resistance to blast disease.

## 2. Results

### 2.1. Exploratory Data Analysis and Overview of the Analyzed Rice RNA-Seq Data

**Figure 1:**
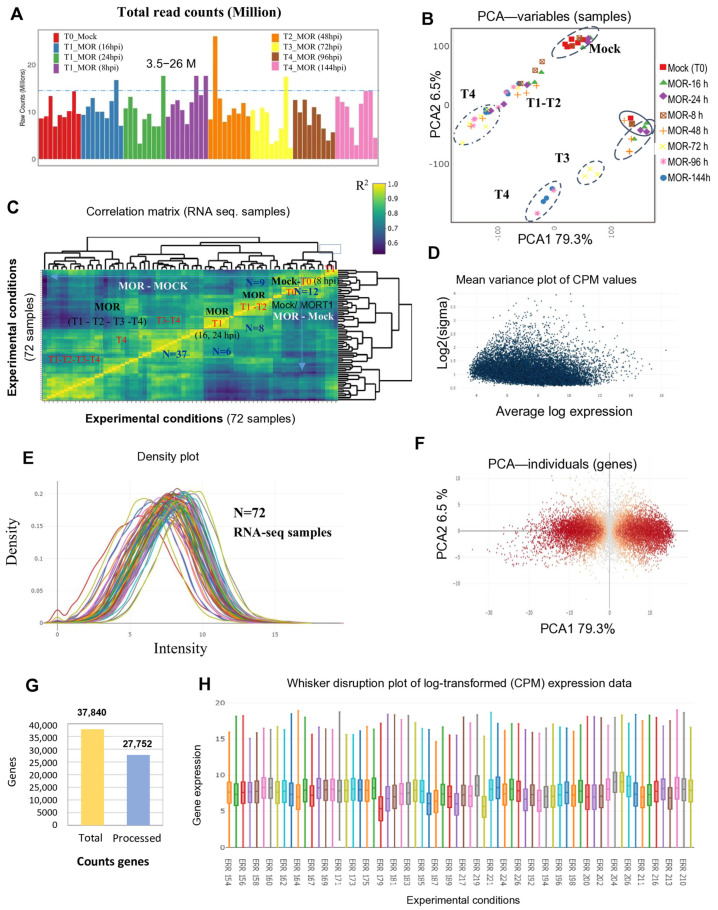
Overview and exploratory analysis of 72 RNA sequencing samples of rice transcriptome profiles infected with MOR at different time courses. (**A**): Total read counts of all analyzed samples. (**B**,**F**); principal component analysis (PCA) revealed differences among samples (**B**) and in eigenvalues for individual (**F**) genes across the top two principal components. The separation of genes based on their first two principal components is illustrated (**F**), where red points indicate genes with highly significant separations, and grey points indicate genes with low significant separations. (**C**): Pearson’s correlation coefficients between samples indicated variability between groups and among replicates within the same group. All biological replicates for the same sample group exceeded 0.9 R^2^. (**D**): The mean–variance relation of CPM values illustrates a flat trend. (**E**): The density plot illustrating the distribution of log-CPM values exhibited a nearly unimodal distribution for each sample. (**G**): Number of genes mapped by type (CPM cutoff of ≥0.5). (**H**): A distribution plot of CPM values illustrates high variation between samples.

RNA-seq raw data from the retrieved sets were analyzed individually, followed by an overview of exploratory data analysis on all the datasets. Read counts from 201 RNA-seq samples were processed, yielding sequencing depths ranging from 2,342,996 to 26,054,301 reads, with an average sequencing coverage of 9,381,439 reads (Figure 1A; Supplementary Data S4). Initially, 37,840 aligned genes were identified across all experiments, with 27,752 mapped genes retained after applying a counts per million (CPM) cutoff filter of ≤0.5 (Figure 1G). Before conducting differential gene expression analysis, the RNA-seq data were evaluated for biological relevance, overall quality, and reliability. Principal component analysis (PCA) and Spearman’s correlation matrix revealed clear distinctions between MOR-infected and mock-treated groups (Figure 1B,C; Supplementary Figure S1). PCA further highlighted variations in eigenvalues for individual genes (Figure 1F) and samples (Figure 1B) across the top two principal components, PC1 (79.3%) and PC2 (6.5%), providing discrimination between the MOR-infected and mock groups (Supplementary Figure S1C,F,I,L). Pearson’s correlation analysis further highlighted variability between MOR-infected and mock groups (Figure 1C; Supplementary Figure S1A,D,G,J). R^2^ values between the MOR and mock groups predominantly ranged from 0.7 to 0.8, while almost all the biological replicates within the same group achieved R^2^ values exceeding 0.9 (Figure 1C; Supplementary Figure S1A,D,G,J; Supplementary Data S9 and S10). The density distribution of log-CPM values demonstrated a nearly unimodal distribution for each RNA-seq sample (Figure 1E and Supplementary Figure S1B,E,H,K), indicating that most samples exhibit a near normal distribution, thereby supporting the overall quality and uniformity of the data. Furthermore, the mean–variance relationship of CPM values exhibited a flat trend, with variance values plateauing at high expression levels (Figure 1D), suggesting that genes with lower expression levels showed greater variability across samples, while higher expression levels exhibited more consistent gene expression profiles.

### 2.2. Exploratory Data Analysis and Overview of the Rice Microarray Data

**Figure 2:**
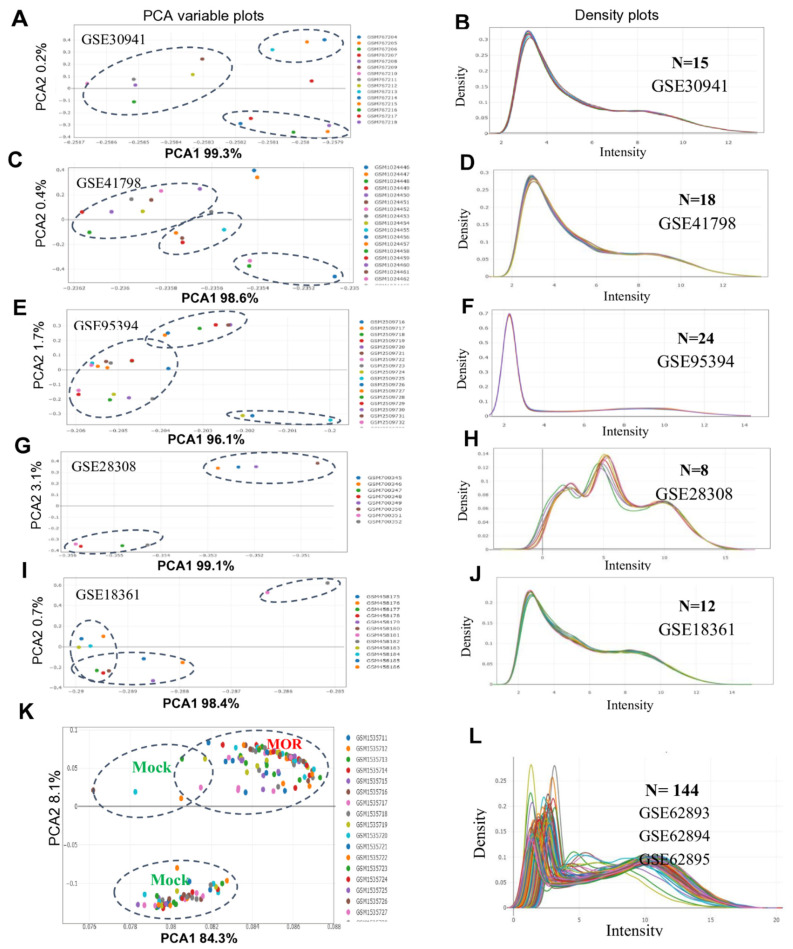
Overview and exploratory data analysis of the rice microarray samples. (**A**,**C**,**E**,**G**,**I**,**K**): Principal component analysis (PCA) variable plots. PCA revealed differences among experimental groups (MOR-infected/mock samples) via the top two principal components (PCA 1 and PCA 2). (**B**,**D**,**H**,**F**,**J**,**L**): Expression density plots illustrating the distribution of the expression values of genes in the dataset. Plot density curves appear to be nearly normally distributed in GSE30941, GSE41798, GSE95394, and GSE18361, while plot density curves appear not to be normally distributed and require further normalization before the screening for differentially expressed genes in GSE28308, GSE62893, GSE62894, and GSE62895 expression profiles.

Exploratory data analysis was conducted for the expression profiles of 221 microarray samples from the Affymetrix GPL2025 and GPL6864 datasets (Figure 2; Supplementary Figures S2 and S3). The GPL2025 expression datasets generally exhibit a highly structured and homogeneous expression pattern, while the GPL6864 dataset shows more diverse expression patterns. PCA analysis revealed that PC1 accounted for 96.1% to 99.3% of the total variance in GPL2025 datasets, with PC2 capturing only 0.2% to 3.1% (Figure 2A,C,E,G,I). However, in the GPL6864 dataset, PC1 explained 84.3% of the variance, while PC2 showed 8.1%, indicating higher multidimensional variability within the GPL6864 profiles (Figure 2K). The density distribution of log-transformed values across 144 samples of the GPL6864 platform (GSE62893, GSE62894, and GSE6295) showed a bimodal distribution, which required forcing normalization before the differential gene expression analysis (Figure 2L). However, the expression profiles of the Affymetrix GPL2025 array sets (GSE30941, GSE41798, GSE95394, and GSE18361) generally exhibited predominantly unimodal distributions, except for GSE18361, which showed a bimodal pattern (Figure 2B,D,F,H,J). Additionally, the correlation matrix between samples indicated variability between both groups and among replicates within the same group (Supplementary Figure S2). In the GPL6864 expression profiles, the distance correlations between the MOR and mock groups varied, with R^2^ values ranging from 0.6 to 0.8 (Supplementary Figure S2E,F; Supplementary Data S11). In contrast, for the experimental profiles of the Affymetrix GPL2025 array sets, R^2^ values between the mock and MOR groups ranged from 0.8 to 0.9 (Supplementary Figure S2A–D; Supplementary Data S12). Additionally, all the microarray profiles exhibited a mean–variance relationship of CPM values with a flat trend, where variance values plateaued at high expression levels, suggesting consistent gene expression profiles for the highly expressed genes (Supplementary Figure S3A,C,G,I,K).

### 2.3. Transcriptional Landscape of Rice PTI-Related Genes upon MOR Infection

**Figure 3:**
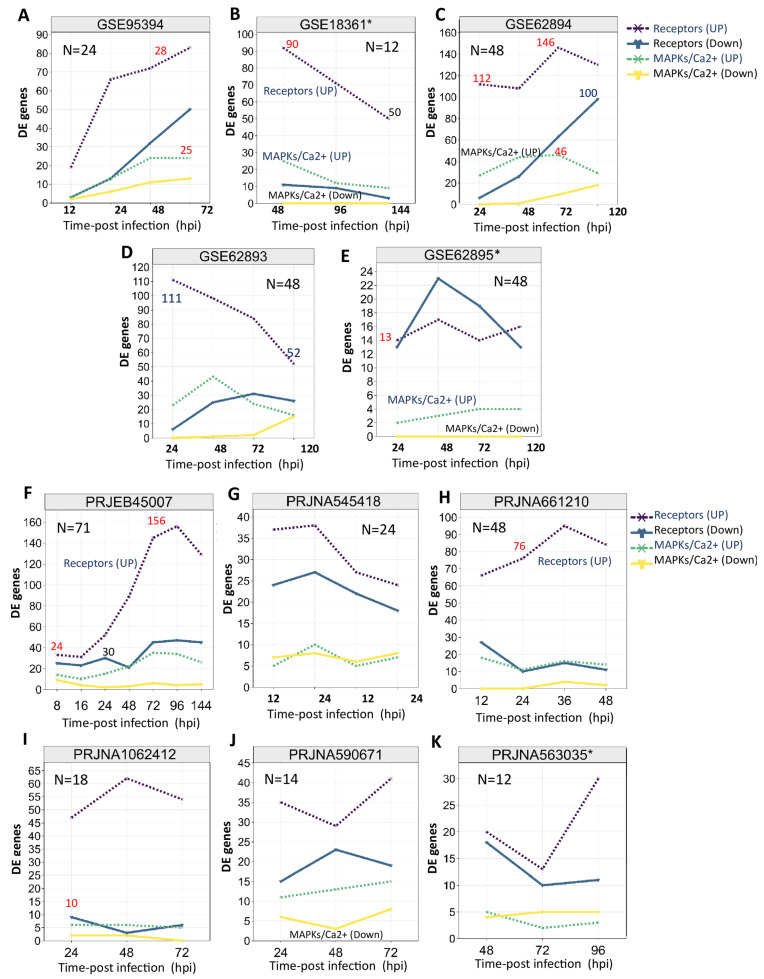
Transcriptional trends of the induced and repressed receptor-based genes and MAPK/Ca^2+^ downstream signaling intermediates form the analyzed RNA-seq and microarray datasets infected by MOR. (**A**–**E**): Line graphs showing the number of induced and repressed genes in the rice microarray datasets. (**F**–**K**): Line graphs showing the number of induced and repressed genes in the rice microarray datasets. All the data are based on Table 1. The dashed lines indicate the induced receptor-based genes (dark purple color) and MAPK/Ca^2+^ genes (green color). The solid lines indicate the repressed receptor-based genes (dark blue color) and MAPK/Ca^2+^ genes (yellow color). “hpi” indicates hours post-infection (GSE18361 * and GSE62895 *: root samples, PRJNA563035 *: spike samples).

In this study, we identified the transcriptional dynamics of the receptor-like kinase genes and downstream factors involved in rice PTI in response to MOR infection. A comprehensive analysis of RNA-seq and microarray data revealed distinct expression patterns of the rice surface receptors and downstream signaling genes across various experimental conditions (Table 1; Figure 3; Supplementary Data S5 and S6). Notably, the rice extracellular receptors and downstream signaling genes, including MAPK and Ca^2+^ signaling genes, were consistently induced in response to MOR infection across all the transcriptomic profiles. Furthermore, most of these genes showed increased expression during pathogen infection rather than a decrease (Table 1; Figure 3).

In the microarray expression profiles, the transcriptional patterns of receptor-based genes and downstream signaling genes revealed dynamic regulatory changes in response to MOR infection (Table 1; Figure 3A–E). A total of 207 microarray datasets from two Affymetrix rice platforms (GPL6864 and GPL2025) were analyzed. The receptor-based genes showed a transcriptional trend, with induced genes ranging from 13 to 146 across different experimental conditions of the rice microarray profiles, while a pattern of one to ninety-eight repressed genes was observed (Table 1; Figure 3A–E). Similarly, MAPK/Ca^2+^ signaling genes exhibited transcriptional trends, with induced genes ranging from two to forty-six, while the number of repressed genes fluctuated from zero to eighteen across the experimental profiles. For instance, GSE62894 from the Affymetrix-GPL6864 dataset exhibited a higher transcriptional abundance, with 112 to 146 extracellular/cytoplasmic receptor genes being induced across different infection time points (Table 1; Figure 3C). However, the GLP2025 profiles (GSE30941, GSE95394, GSE41798, GSE18361, and GSE28308) showed a fluctuating transcription trend of 13 to 92 induced genes at different infection time points (Table 1; Figure 3A,B). Interestingly, rice root transcriptomic data from GSE18361 and GSE62895 profiles highlighted the transcriptional abundance of receptor- and MAPK/Ca^2+^ signaling-related genes in response to MOR infection (Table 1; Figure 3B,E). A total of 14 to 92 (GSE18361) and 14 to 17 (GSE62895) receptor genes were induced across infection time points. Remarkably, no repression was observed for MAPK/Ca^2+^ genes in either the rice root-transcriptomic dataset during MOR infection (Table 1; Figure 3B,E), suggesting a potential constitutive signaling response in rice roots during MOR infection.

Across all the microarray profiles, the transcriptional trends of receptor-based genes fluctuated at different infection time points (Figure 3). Generally, during the early stages of MOR infection (12 to 24 hpi), the transcription abundance of receptor genes ranged from 13 to 112 induced genes, with a pattern of 2 to 27 MAPK/Ca^2+^ signaling genes induced at the same time (Figure 3). The transcriptional activation of both receptor-based genes and MAPK/Ca^2+^ signaling almost peaked around the 48 and 72 hpi, with a maximum of 146 receptor genes and 16 signaling genes induced (Figure 3A,C,E). Notably, receptor genes exhibited a near peak of transcriptional repression as the infection progressed, particularly rice leaf transcriptomic profiles (Figure 3A,C,D). In contrast, rice root transcriptomic profiles showed gradual transcription repression of these genes as the infection advanced (Figure 3B,E), highlighting the complex regulatory mechanisms of these genes during MOR infection across different tissues.

Across the analyzed RNA-seq datasets, receptor and MAPK/Ca^2+^ signaling genes displayed varying transcriptional trends across infection stages. Analysis of 201 RNA-seq samples from eight distinct bio-projects (Table 1) revealed that the receptor-based genes generally exhibited higher activation levels than repression across all experimental conditions (Table 1; Figure 3F–K). The transcriptional patterns of receptor and MAPK/Ca^2+^ signaling genes across different infection stages exhibited dynamic fluctuations in both induced and repressed gene counts (Figure 3). During the early stages of MOR infection (8, 16, and 24 hpi), the number of induced receptor genes ranged from 24 to 76, while a total range of 10–30 genes was repressed. The transcriptional activation of MAPK/Ca^2+^ signaling genes during early infection ranged from five to eighteen induced genes, with repressed gene counts fluctuating between zero and eight (Table 1). In the mid-infection phases of MOR infection (36, 48, and 72 hpi), the receptor genes peaked with 145 induced genes at 72 hpi, while the number of repressed genes fluctuated, reaching a maximum of 45 (Figure 3F). Furthermore, the transcription level of receptor genes across 72 RNA-seq samples almost peaked at the late stages of MOR infection (96, 120, and 144 hpi), with up to 156 genes induced at 96 hpi (Figure 3F).

Overall, the complex regulatory patterns of receptor-based genes and signaling intermediates that vary across the tissues and the experimental conditions of both the microarray and RNA-seq profiles may underscore the intricate interplay between PTI mechanisms in rice response against MOR infection.

### 2.4. Transcript Dynamics of Robust Extracellular and Cytoplasmic Receptor-Based Genes

We further illustrated the transcript dynamics in response to MOR by monitoring the robust number of increased and reduced genes commonly expressed across the RNA-seq and microarray datasets. Data were organized into four time courses of each RNA-seq and microarray expression profile: T1 (8–24 hpi), T2 (36–48 hpi), T3 (72 hpi), and T4 (96–144 hpi). The robust rank aggregation (RRA) algorithm was employed to consolidate multiple expression profiles derived from individual experiments of the RNA-seq and microarray datasets. The robust induced and repressed genes were extracted from each dataset pattern (RNA-seq/microarray). The robust genes at each time point were algorithmically generated compared to a baseline distribution of log_2_fold changes across the entire DEG lists.

**Figure 4:**
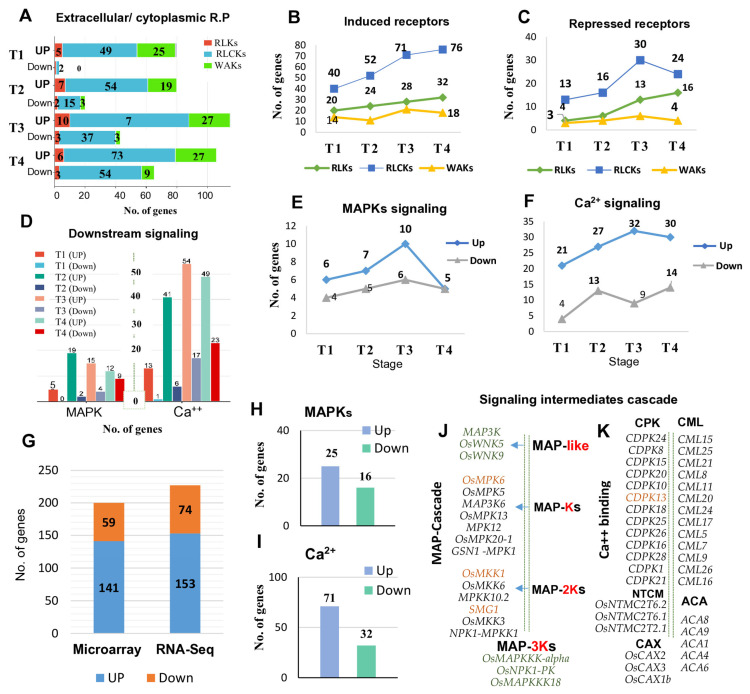
Transcript profile of robust receptor-based genes in the RNA-seq and microarray dataset in response to MOR infection at different stages. (**A**–**C**): Transcriptional level of extracellular and cytoplasmic receptors, including RLKs, WAKs, and RLCKs during infection. (**A**): The number of induced and repressed receptor genes in the microarray profiles. (**B**): The number of induced genes in RNA-seq profiles. (**C**): The number of repressed genes in the RNA-seq profiles. (**D**–**F**): Transcript dynamics of downstream gene groups, including Ca^2+^ and MAPKK. (**D**): The number of induced and repressed MAPKs and Ca^2+^ downstream signaling genes in microarray profiles. (**E**,**F**): The number of induced (blue line) and repressed (grey line) MAPK (**E**) and Ca^2+^ (**F**) signaling genes in the RNA-seq profiles. (**G**): Total number of activated and repressed receptor genes in both the RNA-seq and microarray profiles. (**H**,**I**): Total number of activated and repressed MAPK/Ca^2+^ downstream intermediate signaling genes in both RNA-seq and microarray profiles. (**J**): A simple diagram illustrating the most frequently activated key genes of the MAPK phosphorylation/activation cascade (*MAPKs*, *MAPKKs*, *MAPKKKs*, and MAP-like kinase genes), across MOR-infected profiles. (**K**): Frequently, key observed Ca^2+^ signaling genes (including CPKs, CMLs, CAX, ACA, and NTCM) were induced across profiles.

The results showed that the number of receptor-based genes with altered transcripts increased as the infection time progressed in both the RNA-seq and microarray profiles (Figure 4A–C,G; Supplementary Data S7). Receptor-like cytoplasmic kinases (RLCKs) exhibited high transcript dynamics in response to MOR infection. Initially, at T1 stage, 40 robust RLCK genes were induced in the RNA-seq profiles (derived from 60 samples), and 47 genes were induced in the microarray profiles (derived from 52 samples), increasing to 76/78 genes at T3/T4 based on a total of 47 RNA-seq and 35 microarray samples (Figure 4B,C). In contrast, two to fifty-four genes were repressed in all the microarray profiles (Figure 4A), whereas fourteen to thirty genes were repressed in all the RNA-seq profiles (Figure 4C). It seems that more RLCKs are needed to battle against the pathogen’s expansion in rice plants.

Notably, both wall-associated kinases (WAKs) and receptor-like kinases (RLKs) also exhibited remarkable responses to MOR infection, although the number of robustly induced and repressed genes was lower than in RLCK genes across all time points. The transcript abundance dynamics of WAKs and RLKs showed relative oscillations across the RNA-seq and microarray profiles (Figure 4A–C). Across the RNA-seq profiles (T1–T4), 20–32 *RLK* genes were robustly induced, compared to 5–10 genes in the microarray profiles (Figure 4A,B). However, 25–27 WAK genes were induced in the microarray profiles (Figure 4A), compared to 14–18 genes in RNA-seq profiles (Figure 4B). By combining all the robust genes for each transcriptomic data across all time points, we found that a maximum of 141–153 receptor genes were induced in the microarray and RNA-seq datasets, while 59 to 74 genes were repressed (Figure 4G).

### 2.5. Top Induced Receptors in Rice Response to MOR Infection

**Figure 5:**
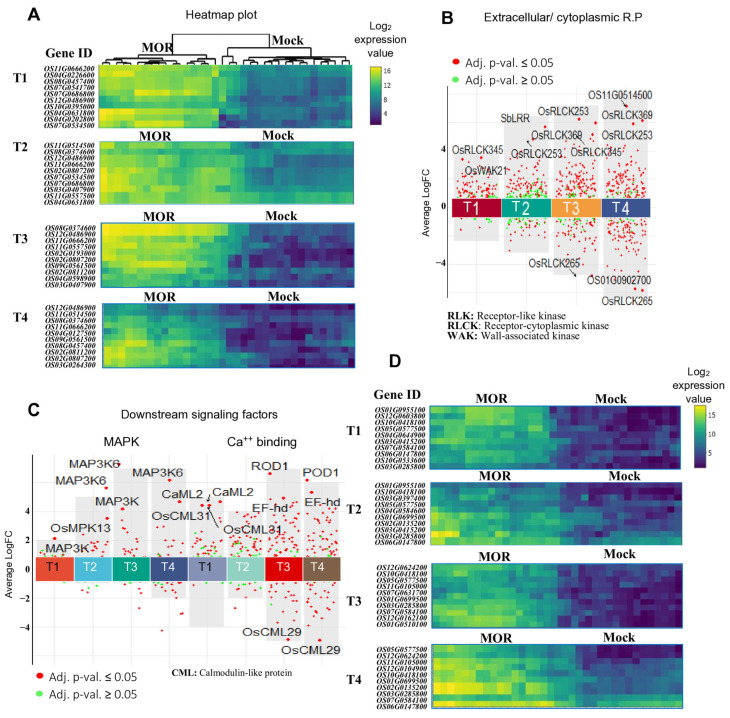
Expression profiles of top activated genes of the extracellular/cytoplasmic and signaling intermediates across the different infection stages. (**A**): Heatmap plots illustrate the expression profiles of the top ten activated receptors across MOR-infected and healthy conditions across the four time points. Each time point was derived from 18 paired samples (MOR vs. Mock). Scatter plots illustrating the expression profiles of the extracellular/cytoplasmic receptors (**B**) and downstream factors (**C**), in a set of 144 microarray samples (Affymetrix GPL6864 platform). Each point represents a gene; significantly regulated genes (adj. *p*-val. ≤ 0.05) are marked in red, while non-significantly regulated genes (adj. *p*-val. ≥ 0.05) are marked in green. (**D**): Heatmap plots illustrate the expression profiles of the top ten activated signaling intermediates (Ca^2+^/MAPK) genes across the MOR-infected and healthy conditions across the four time points.

We identified the top consistently induced receptor-based genes across all the analyzed transcriptomes (Table 2). Notably, *OsRLCK255* (*Os08g0457400*) was frequently most induced in 190 MOR-infected samples (72 microarrays/122 RNA-seq) across the four infection time courses. Among the wall-associated receptors, WAK20 and WAK24 were the most frequently top-induced genes, appearing in 126 (66%) and 158 (83%) MOR-infected samples, respectively (Table 2). Additionally, two Lysin-motif extracellular domain-containing RLK genes (*OsLysM-RLK7* and *OsLysM-RLK1*) were top frequently upregulated in 54 and 18 samples, respectively (Table 2). All the top activated genes exhibited a notable increase in expression levels (log_2_-scale) in the MOR-infected samples compared to the mock (healthy) samples (Figure 5A). Furthermore, analysis of 144 microarray samples revealed that the significantly expressed extracellular and cytoplasmic genes tended to increase their transcription as the MOR infection time progressed (Figure 5B).

### 2.6. Common Robust Receptor-Based Genes in Rice Response to MOR Infection

**Figure 6:**
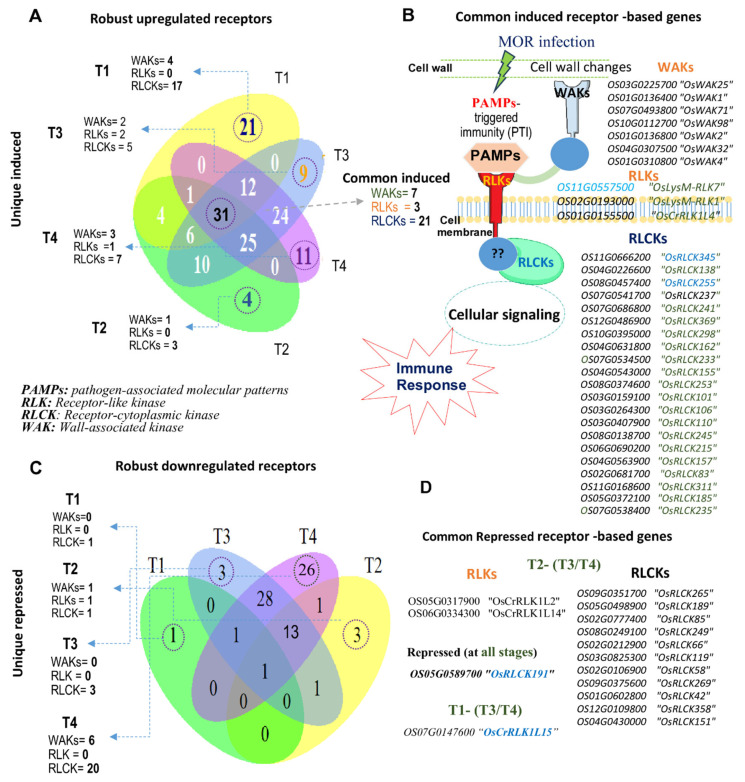
Transcript dynamics of extracellular and cytoplasmic receptors across all the rice transcriptome data. (**A**): Venn diagram illustrating the consistently activated receptors across the RNA-seq and microarray profiles during the four stages of MOR infection. (**B**): A common set of 31 activated receptors across all the infection stages. Among these, WAKs (wall-associated receptors) are cell wall-embedded and transmembrane receptors that may recognize any extracellular changes derived either from pathogen-associated molecular patterns (PAMPs) or host damage-associated molecular patterns (DAMPs). RLKs (receptor-like kinases) are central transmembrane proteins involved in the first lines of PTI machinery, initiating signaling cascades by PAMP recognition. RLCKs (receptor-like cytoplasmic kinases) are intracellular signaling molecules often associated with transmembrane proteins and lack extracellular domains. (**C**): Venn diagram showing the number of repressed receptor genes during different infection stages. (**D**): Commonly repressed receptors across all time points during infection. Only one receptor was repressed in common across all profiles (*OsRLCKs191*, *Os05g589700*). The number of RLCK genes with altered expression was higher than other receptors across all dynamic profiles.

We further provide an overview of the commonly induced and repressed genes across all the transcriptomic profiles at four different time courses (Figure 6A–D). The integrated responses from multiple receptors were identified including three RLKs (*OsLysM-RLK7*, *OsLysM-RLK1*, and *OsCrRLK1L4*); seven WAKs (*OsWAK25*, *OsWAK1*, *OsWAK71*, *OsWAK98*, *OsWAK2*, *OsWAK32*, and *OsWAK4*); as well as 17 *RLCK* genes, including *OsRLCK345*, *OsRLCK162*, *OsRLCK245*, *OsRLCK185*, *OsRLCK83,* and others (Figure 6A,B). The potential integration among the robust RLK/RLCK and RLCK may be synergized under MOR infection, demonstrating the intertwined genetic network in rice immune responses to MOR infection (Figure 6B). Conversely, among the robust downregulated receptor genes, only the *OsRLCK 191* “*Os05g0589700*” gene was repressed across all the transcriptomic time points (Figure 6C,D). Overall, the consistent induction of these genes across all infection stages suggests their multifaceted roles as central components of the rice defense response, contributing to a dynamic and sustained resistance against MOR infection. These robust common genes may serve as genetic hubs for breeding strategies, providing informative clues that can be exploited to enhance signaling-dependent mechanisms contributing to durable and broad-spectrum resistance in rice against blast disease.

### 2.7. Stage-Specific Transcription of Receptor-Based Genes in Rice Response to MOR

**Figure 7:**
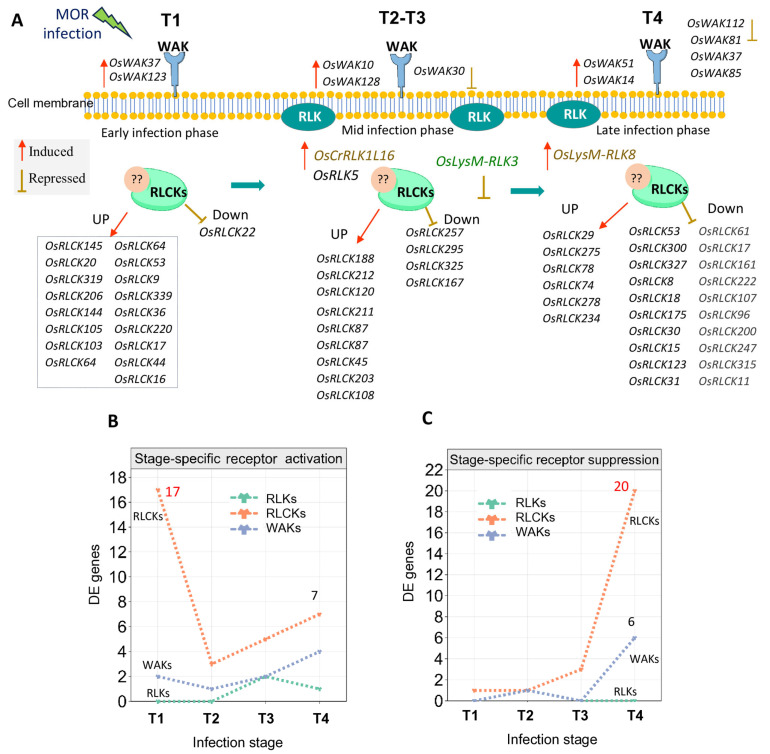
Stage-specific activation and repression of receptor genes intimately respond to MOR infection at a specific time point. (**A**): Model diagram of the unique activated and repressed genes at each infection stage (T1, T2, T3, T4). The number of RLCK genes with altered expression was higher than other receptors across all dynamic profiles. At the early infection phase, only two extracellular WAK genes (*OsWAK37* and *OsWAK123*) were specifically activated, followed by the activation of 17 cytoplasmic receptor genes (RLCKs), whereas no specific extracellular receptors of the RLK family were activated. At mid-infection phase, two specific extracellular genes of RLKs and two genes of WAKs were activated, and nine RLCKs were specifically activated. At the late infection stage, specific RLCK genes with altered repression were observed, with 20 repressed genes. (**B**,**C**): Line graphs showing the number of unique induced (**B**) and unique repressed (**C**) receptor-based genes at each time point. RLKs indicate receptor-like kinase genes, RLCKs indicate receptor-like cytoplasmic kinase genes, and WAKs indicate wall-associated kinase genes. All the unique induced and repressed are based on Table 3. The T1 stage indicates 8-, 12-, 16-, and 24-h post-infection (hpi), the T2 stage indicates 36–48 hpi, the T3 stage indicates 72 h, and the T4 stage indicates 96, 120, and 144 hpi.

The robust aggregation algorithm analysis across all RNA-seq and microarray datasets also highlighted distinct robust receptor genes uniquely induced or repressed at specific stages of MOR infection across all the DEG profiles (Figure 7A–C; Table 3). Inimitably, several unique genes were induced (Figure 7B) and repressed (Figure 7C) at specific time points, indicating their distinct response to MOR infection at different infection stages. At the T1 stage, a notable transcriptional activation of RLCK genes was observed, with 17 unique genes induced, including *OsRLCK145*, *OsRLCK20*, and other RLCK genes (Table 3; Figure 7A,B). Additionally, two unique WAK genes were induced (*OsWAK37* and *OsWAK123*), while no RLK genes were specifically expressed at this stage (Figure 7). This suggests the early and dominant role of RLCKs in the initial stages of rice responses to MOR infection. At both the T2 and T3 stages of MOR infection, WAKs and RLCKs exhibited limited stage-based infection transcription compared to the T1 stage (Table 3; Figure 7). For instance, one WAK gene (*OsWAK10*) and three RLCK genes (*OsRLCK188*, *OsRLCK212*, and *OsRLCK120*) were specifically induced at the T2 stage profiles (Figure 7A,B). This specific transcription alteration of receptor genes across the early and mid-stages of MOR infection may indicate a kind of reconfiguration of receptor-mediated signaling pathways after the initial response. Furthermore, two WAKs (*OsWAK128* and *OsWAK102*), two RLKs (*OsRLK5* and *OsCrRLK1L16*), and five RLCKs were uniquely induced at all the T3 stage profiles (Table 3; Figure 7A,B). At the late infection stage (T4 profiles), three WAKs and seven RLCK genes were specifically induced, while one RLK gene (*OsLysMRLK8*) was induced, suggesting the specific role of these genes in sustaining the defense responses to MOR infection at late stages.

Conversely, a total of 20 RLCK and 6 WAK genes were specifically repressed at the late stage of the MOR infection stage (Figure 7; Table 3). Overall, our results underscore the dynamic and robust stage-specific transcriptional activation/suppression patterns of receptor-based kinase families in rice during the MOR infection. The complex regulatory patterns of these genes highlight an intricate interplay in rice responses, particularly within the PTI mechanism and its signaling intermediate.

### 2.8. Transcript Dynamic and Scaffolding Profile of Robust Downstream Signaling Intermediates in Response to MOR Infection

Downstream signaling factors, including mitogen-activated protein kinase (MAPK) and calcium (Ca^2+^)-mediated signaling pathway genes, exhibited notable responses to MOR infection (Figure 4D–F,H–K; Supplementary Data S8). The transcript abundance dynamics of MAPKs and Ca^2+^ sensors increased steadily as infection time progressed (Figure 4D–F). Across microarray data, five to nineteen MAPK robust genes were induced, compared to two to nine genes that underwent repression (Figure 4D). Simultaneously, genes involved in calcium-mediated signaling pathways were upregulated, with 13–54 genes induced and 1–23 genes repressed out of the 83 annotated Ca^2+^-related genes.

In the RNA-seq profiles, the numbers of induced and repressed MAPKs exhibited a relatively consistent pattern. Approximately six robust genes were upregulated at the T1 stage, reaching a peak of ten genes at the T3 stage (Figure 4E). Meanwhile, 4–6 MAPK genes were repressed across all time points (Figure 4F). Ca^2+^-related genes exhibited a prominent response to MOR infection (Figure 4F). A robust set of 16 genes was activated at the T1 stage, followed by 27, 33, and 30 upregulated genes in RNA-seq profiles at the subsequent time points. Conversely, four genes were consistently repressed at the T1 stage, reaching a maximum of 14 repressed genes at T4.

A total of 26 *MAPK* genes were induced across all the transcriptomic profiles, compared to 16 repressed genes (Figure 4H). Transcript patterns of Ca^2+^-related genes showed 71 induced genes, while 32 genes were repressed (Figure 4I). Among the 71 induced genes, 58 frequently activated genes belonged to different calcium sensor-binding proteins, including calcium-dependent protein kinase (CDPK), calmodulin-like proteins (CaMLs), vascular cation exchanger proteins (CAXs), calcium transporting ATPase (ACA), and N-terminal TM-C2 proteins (NTCMs). Additionally, 13 genes encoded EF-hand domain proteins (Supplementary Data S8).

We further illustrated the scaffolding profile of MAPKs and MAPK-like proteins for the most frequently regulated genes in rice response to MOR infection (Figure 4J; Supplementary Figure S4). Across the MAPK cascade, two MAPK-like genes, *MAP3K6* and *OsWNK5* (With no Lysine Kinase), were commonly observed in all the transcriptomics time points. Similarly, two MAPK genes (*MPK6* and *MSRMK2*) and two MAPK(K) genes (*OsMKK1* and *SMG1-OsMAPKK4*) showed consistent upregulation across all the transcriptomes under MOR infection (Figure 4J; Supplementary Figure S4).

### 2.9. Top-Induced Signal Intermediates in Rice Response to MOR Infection

We identified top activated signaling intermediates, including Ca^2+^ influx and MAPK scaffolding genes, which exhibited high frequency across all the transcriptomic data analyzed (Table 4). Among calcium-related genes, calmodulin-like protein-encoding genes (CaMLs) were the most frequently top-induced genes across all the time points. Specifically, *CaML31* and *CaML5* genes were frequently identified in 176 and 131 MOR-infected samples, respectively (Table 4). The Ca^2+^-dependent protein kinase (*OsCDPk13*) gene was frequently most activated at the T1-T3 profiles in 60 MOR samples. However, the *OsNTMC2T2.1* gene with an N-terminal transmembrane was top-induced at the T1 stage in 18 microarray samples.

Regarding MAPK gene activation, MAP kinase-like protein genes, including *MAP3K* (*OS03G0415200*) and *OsWNK5* (*OS07G0584100*), were observed among the top activated genes in 117 and 81 MOR-infected samples, respectively. *OsMKK1* (*OS06G0147800*), a MAPK(K) gene, was frequently most induced in 120 samples. Among all MAPK genes, *OsMSRMK2* (*OS03G0285800*) was consistently most activated across all the infection stages in 176 MOR-infected samples (Table 4).

Furthermore, the transcriptional profile of the top 10 upregulated genes across different infection stages was illustrated in a set of microarray samples to show the discrimination in the expression level of these genes under MOR-infected and mock-health conditions (Figure 5D). Likewise, scatter plots of robust MAPK and Ca^2+^-mediated downstream signaling genes derived from a total set of 144 microarray samples showed the transcriptional trend of significantly expressed genes across the infection stages (Figure 5C).

### 2.10. Key Interconnected Genes Among the Extracellular/Cytoplasmic Receptors and Signaling Intermediates in Rice Response to MOR Infection

**Figure 8:**
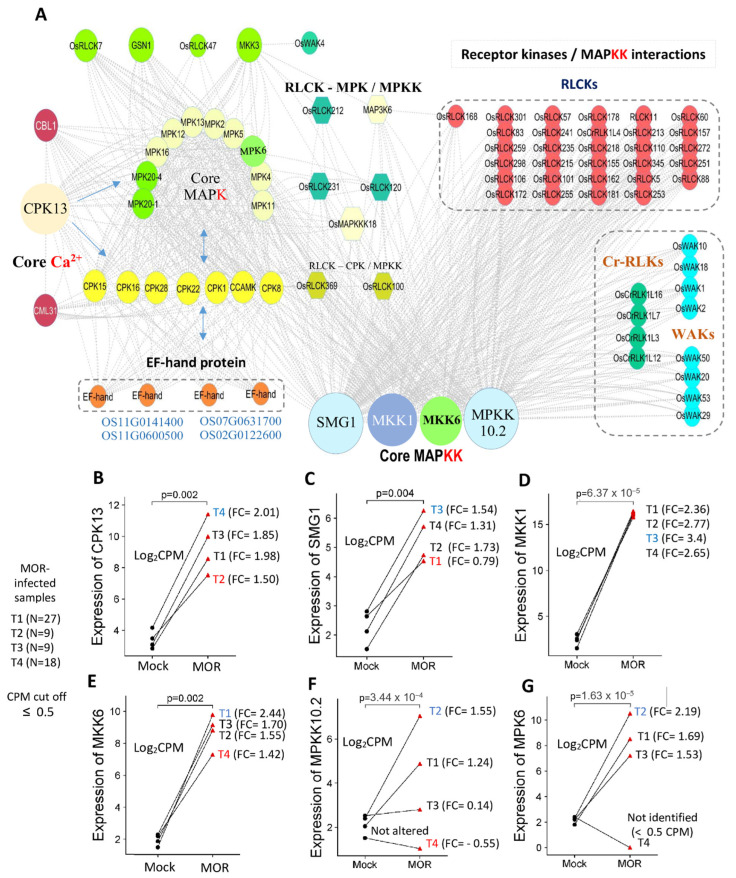
Key interconnected genes among the extracellular/cytoplasmic receptors and signaling intermediates in rice response to MOR infection. (**A**): Protein–protein interaction networks among key response gene groups. Extracellular receptors (RLKs, WAKs), cytoplasmic receptor kinase (RLCK), and downstream cascades of MAPKs and Ca^2+^ binding. Among Ca^2+^ sensors, eight CPK genes (calcium-dependent protein kinase) were highly connected to MAPKs, MAPKKs, and EF-hand proteins. Ten core *MAPK* genes were found to be connected with CPKs and MAPKKs. The four top-connected genes associated with MAPKKs were observed, including *SMG1*, *MKK1*, *MKK6*, and *MPKK10.2*. Four EF-hand protein genes were mapped and connected to core Ca^2+^ and *MAPK* signaling genes. (**B**–**G**): Expression profile of six core MAPK/Ca^2+^ intermediate genes across 72 RNA-seq samples.

To elucidate the complex interactions among key genes, we constructed a PPI map across all the time points, highlighting core genes among/between extracellular/cytoplasmic receptors and downstream signaling factors, including Ca^2+^ sensors and MAPKs (Figure 8A). Our analysis revealed diverse interactions between core genes from various protein kinases (MAPKs, MAPKKs), Ca^2+^ sensors, and receptor kinases (RLKs, RLCKs, WAKs).

Interestingly, our analysis revealed strong network interaction among four MAPKKs (Mitogen-Activated Protein Kinase Kinases) genes (*SMG1*, *MKK1*, *MKK6*, and *MPKK10.2*), along with forty-four receptors (thirty-two RLCKs, four RLKs, and eight WAKs) and other MAPK genes (Figure 8A). The connections between these MAPKK and receptor-based genes imply a complex role in the rice immune signaling response to MOR infection.

In response to MOR infection, the CPK13 gene emerged as a top Ca^2+^-related core gene, connecting to several MAPK genes and Ca^2+^ sensors. Approximately 11 protein kinase genes (MAPKs) were highly connected to CPK genes and other MAPKK genes. Additionally, two RLCKs (*OsRLCK231*, *OsRLCK120*) were interconnected with both MAPKs and MAPKKs, while another two (*OsRLCK100*, *OsRLCK369*) were mapped in connecting with CPK genes and MAPKK genes. Four EF-hand protein genes were mapped and connected to core Ca^2+^-related genes and core MAPK genes (Figure 8A). Taken together, these networks suggest that these signaling intermediates and receptor-based genes may play key roles in orchestrating immune responses to MOR infection in rice.

The expression profiles of six key genes (*SMG*, *MKK1*, *MKK6*, *MPPK10.2*, *MPK6*, and *CPK1*) were analyzed using the log_2_CPM values from 72 high-throughput RNA-seq samples to observe their transcriptional response between MOR-infected and mock-healthy conditions over four time points (Figure 8B–G). *MKK1*, *MKK6*, and *CPK1* genes exhibited consistent transcription activation in the MOR-infected samples compared to mock samples (Figure 8). The highest levels of expression were observed at T3 stage for *MKK1* (log_2_CPM = 16.25 vs. 1.62 (mock), log_2_FC = 3.4), at T1 stage for *MKK6* (15.77 vs. 3.05 (mock), log_2_FC = 2.44), and at T4 stage for *CPK1* (11.81 vs. 2.99 (mock), log_2_FC = 2.01), indicating a robust and sustained response to the MOR infection across different time points (Figure 8B,D,E). Similarly, the *MKK6* gene demonstrated expression alteration, peaked at the T2 stage (log_2_CPM = 16.42 vs. 2.4; log_2_FC = 2.19), though expression slightly decreased as the infection progressed (Figure 8G). *SMG1* gene shows a gradual increase in expression in the MOR-infected group compared to the control, with low alteration at the initial time point (log_2_CPM = 3.88 vs. 2.4 (mock), log_2_FC = 0.79) (Figure 8C). Overall, the MOR infection induces a varied response across the core genes, where *MKK1* and *CPK1* showed stable upregulation, while genes such as *MPPK10.2* and *MPK6* demonstrated more dynamic fluctuations.

## 3. Discussion

### 3.1. Overview and Exploratory Data Analysis of Rice RNA-Seq and Microarray Transcriptomic Data

Over the past two decades, RNA-seq and microarray-based studies have revolutionized our understanding of genome-wide transcriptomic changes, opening new avenues for investigating plant–pathogen interactions. Despite the wealth of data generated from these studies, a significant amount of information remains untapped, warranting further exploration and analysis. A central question guiding our integrative analysis is how to effectively combine and analyze the data from multiple existing RNA-seq and microarray studies conducted under *Magnaporthe oryzae* (MOR) infection conditions to assess the transcriptomic profiles of receptor-based genes and identify their responsiveness to pathogen infections.

Comparing gene lists across different studies can be challenging due to variations in datasets. Technical variations in RNA-seq/microarray methods, such as library preparation, sequencing method, as well as the bioinformatics data analysis pipeline, contribute to these challenges. To overcome these limitations, we employed linear modeling combined with empirical Bayes moderation (eBayes) for differential gene expression (DGE) analysis across both approaches, ensuring more accurate results and minimizing variability between RNA-seq and microarray datasets [30,31]. Using a consistent bioinformatics pipeline and analysis criteria for all the experimental conditions increases the robustness and reliability of output findings, notably in DEG selection.

Our exploratory data analysis dimensionality indicated an overview of the structure and relationships among the analyzed samples in all the experiments (Figure 1 and Figure 2; Supplementary Figures S1–S3; Supplementary Data S9–S12). Our results suggest the constitutive differences between experimental groups [32]. RNA-seq and array sets mostly showed a quite flat trend between the means and variances (Figure 1F and Figure 2A,C,E,G,I,K), reflecting high biological variation between experimental conditions [31].

### 3.2. Transcriptional Landscape of Receptor-Based Genes in Rice Response to MOR Infection

The study showed a significant alteration in transcriptional abundance upon MOR infection across different infection stages (Table 1; Figure 3; Supplementary Data S5 and S6). The differences among the set of expressed profiles are attributed to specific experimental conditions such as genotype, resistance/susceptibility, infected tissues (leaves, roots, or panicles), plant growth conditions, the methods of inoculation, and plant growth stage. Generally, transcriptomic profiles have been widely used to identify specific gene expressions, providing significant insights into the molecular mechanisms underlying plant resistance to pathogens such as MOR, and other significant pathogens that affect rice [33,34,35,36,37,38,39,40,41,42,43,44,45,46,47,48,49,50,51]. Despite extensive research and growing advances in understanding rice immunity to MOR infection, several key aspects of the interplay between rice and MOR remain poorly understood. While previous research has contributed valuable insights into the rice–MOR interaction, it often focuses on specific studies, including particular genes or specific signaling pathways [35,37,38,39,40], overlooking multifaceted pathways such as PTI, and the transcriptional profiles of receptor-based genes in the rice response to MOR infection. During our study, the landscape profile from individual studies provides an overview of our current understanding of receptor-based genes in rice genome reprogramming under MOR infection, justifies their important roles in rice–MOR interactions, and advocates for their incorporation into the plant immune system. This approach offers a comprehensive understanding of the variation in the rice transcriptional landscape of the receptor-based genes during the defense response to MOR.

### 3.3. PTI-Related Genes Were Prominently Induced in Rice Response to MOR Infection

Since individual transcriptional profiles may exhibit distinct patterns, our integrative analysis identified gene sets that were consistently regulated across conditions with a high degree of confidence, as well as those showing opposing expression patterns. Our findings shed light and provide a robust transcriptional pattern of the receptor-based genes and signaling downstream scaffolding that are mainly involved in PTI machinery in rice (Figure 4, Figure 5, Figure 6, Figure 7 and Figure 8; Supplementary Data S7 and S8).

Our results suggest that the transcriptional induction of the extracellular and cytoplasmic receptor genes is prevalent in priming rice immune responses against MOR infection. RLCK receptors were highly responsive to MOR, where the number of expressed genes increased upon infection progression (Figure 4). RLCKs play important roles in triggering plant immunity by acting as intracellular signal transducers, while many of these receptors are still functionally ambiguous [20,52,53]. Likewise, some RLCKs integrate responses from multiple receptors recognizing distinct ligands. Although they lack extracellular domains that exist in other receptor kinases (RLKs and WAKs). However, a previous study showed that *OsRLCK185* interacted with the chitin receptor complex of *OsCERK1* and was phosphorylated by it, initiating downstream signaling in rice immune responses against the MOR infection [19,54].

Our study highlighted that the number of RLCK genes with altered expression was higher than other receptors across all the dynamic profiles (Figure 4A–C,G and Figure 6). Central to these genes, 17 RLCK genes were constantly activated across all the transcriptomic profiles (Figure 6B). Among them, eight genes (*OsRLCK255*, *OsRLCK138*, *OsRLCK345*, *OsRLCK237*, *OsRLCK253*, *OsRLCK233*, *OsRLCK162*, and *OsRLCK106*) were frequently found to be top upregulated across a considerable set of MOR–infected samples (Table 2), highlighting their potential significance in rice signal pathways in response to MOR infection. Furthermore, our results revealed that RLCK genes display stage-specific patterns of activation and repression, playing a critical role in the rice response to MOR infection (Figure 7; Table 3). RLCKs exhibited the most dynamic expression changes across all infection stages. During the early infection phase (T1), 17 RLCK genes were specifically activated, whereas during the late infection stages (T3–T4), RLCK genes were predominantly repressed, with 20 RLCKs specifically downregulated (Figure 7; Table 3). In this manner, it is plausible to suggest that the observed pattern of high transcriptional abundance of *RLCKs* during the early response to *MOR* infection, contrasted with their strong repression at later stages, may be balanced by the suppression of other receptor-related pathways at the late stages of infection. Overall, our results identified that some RLCK genes highly respond to MOR infection and appear to integrate signals from PAMP recognition, suggesting a level of functional specialization of these RLCK genes in rice immunity against MOR infection (Figure 6B).

RLKs and WAKs are extracellular proteins that perceive PAMPs, such as chitin and oligo-glucuronides from fungal cell walls [21,55,56]. Both receptors can coordinate with RLCKs in regulating hormone synthesis and responses, ROS production, Ca^2+^ signaling, activation of MAPK, and immune gene expression. Previous research revealed that 103 genes of these receptors were found to be induced in rice under MOR infection [33]. Our results identified robust transcript-altered genes among both subfamilies of receptors (Figure 4A–C). Additionally, we show that seven WAK and three RLK genes were commonly activated under MOR conditions across all the transcriptomic data (Figure 6B). Wall-associated kinases have been explored as positive and/or negative regulators of rice resistance to fungal blast disease. It has been shown that loss-of-function mutants of *OsWAK14*, *OsWAK91*, and *OsWAK92* positively regulated the quantitative resistance to MOR infection, while *OsWAK112d* acts as a negative regulator of blast resistance [57]. Furthermore, multiple research studies have suggested that WAKs are key regulators of plant disease resistance, as they monitor the integrity of the cell walls and initiate intracellular signaling upon their disruption by pathogen attacks [58]. Genome-edited lines of the *WAK1* gene were found to have impaired callose-induced cell wall reinforcement in response to bacterial flagellin detection [59]. In wheat, the *TaWAK6* gene has been shown to effectively limit pathogen growth, contributing to adult plant resistance against leaf rust disease [60]. Likewise, several studies reported that *the Stb6* gene, which encodes a WAK extracellular protein, was found to recognize a small secreted protein from the fungal pathogens of *Septoria* leaf blotch upon infection [61,62].

In the core of robust WAK and RLK genes, our findings show seven common activated WAK genes and three RLK genes that encode extracellular receptor proteins with lysin-motif domain (Figure 6B). Central to these genes, we show *OsLysM-RLK7* and *OsLysM-RLK1* as top upregulated genes across a set of 55 and 18 MOR-infected samples at the T1/T2/T3 stages (Table 2), while *OsWAK1* gene was most activated across a set of 40 MOR-samples at the T1 and T4 stages (Table 2). The consistent activation of these common genes implies their potential role in initiating rice cellular signaling and immune responses upon MOR invasion. However, the interactions and networks of these receptors in response to both the early and late stages of MOR infection require further investigation to fully reveal their contribution to rice resistance against blast disease.

Based on our findings, the prominent transcriptional activation of these receptors underscores their importance in the downstream signaling of rice immunity against MOR infection. This suggests that PTI may play a crucial role in rice immunity against blast diseases by activating a robust set of extracellular receptors and cytoplasmic receptor-like kinases. However, the suppression of numerous receptor genes in the rice transcriptomes upon MOR infection is also evident (Figure 4G and Figure 6C). Given that receptor kinases are primarily involved in activating resistance mechanisms through PTI, this raises important questions. Specifically, it prompts consideration of whether these receptors might also play a contrasting role in promoting infection, potentially aligning with a phenomenon of PAMP-triggered susceptibility.

### 3.4. Interconnecting Genes of PTI and Downstream Signaling upon MOR Infection

Posttranslational modification of proteins is a crucial regulatory component of all cellular signaling, including plant immune signaling. The transcriptional regulation of plant immune receptors and signaling intermediates may enable rapid adjustment of defense response activation during pathogen infection. In this study, we identified the mode of gene association between extracellular/cytoplasmic receptors and downstream signaling genes (MAPKs and Ca^2+^-signaling genes). As interconnecting core genes, eight WAKs (e.g., *WAK1*, *WAK2*), four RLKs (e.g., *OsCrRLK1L7*), and thirty-two RLCKs show high connectivity with other MAPKK-mediated signaling genes, such as *SMG1, MKK1, MKK6*, and *MPKK10.2* (Figure 8). MAPK cascades are crucial regulators of multiple biological processes in plants, including rice, such as immune responses; however, the actual mechanisms governing their functions are still not fully understood. Among the core genes, *MKK6* and *MPKK10.2* were identified in response to MOR infection (Figure 8). Previous studies revealed that the OsMKK6/OsMPK4 cascade is proposed to regulate rice resistance to MOR, and its disruption has been shown to activate OsMPK6 [63]. Furthermore, OsMPKK10.2/OsMPK6 signaling cascades are suppressed by enhanced disease resistance 1 gene (*OsEDR1*), which plays a role in pre-invasive nonhost resistance, and this suppression functions as a negative regulator of the immune response in rice [64]. OsMKK10.2–OsMPK6 cascade is required to activate the OsWRKY45 transcription factor, leading to salicylic acid-mediated rice disease resistance against pathogen infection [23,64,65]. Additionally, *SMG1* (small grain 1), which encodes OsMKK4, was identified as a core interconnected gene along with *MKK1* (Figure 8). To the best of our knowledge, no prior studies have investigated their roles in rice defense against MOR infection. Therefore, further functional characterization of *SMG1* (*OsMKK4*) and *MKK1* is essential to uncover their potential contributions to rice immunity against MOR.

Generally, kinase-dependent signaling, mediated through PAMPs, receptors, and downstream factors, mainly activates a series of defense responses such as cell death, ROS generation, and defense-related proteins [18,66]. Our findings consistently highlighted the rice RLCKs as key genes responsive to MOR infection, with a considerable set of genes strongly interconnected with multiple MAPK intermediates (Figure 8). RLCK subfamily members have been implicated in playing a crucial role in plant immunity, facilitating strong immune responses by interacting with both PRRs and NLR intracellular receptors. These kinases act as a bridge connecting surface-localized receptors to MAPK signaling cascades and are directly involved in triggering the generation of ROS [67,68]. However, the interactions between receptors and downstream cascades in signaling pathways leading to the activation of defense responses are still poorly understood. Overall, our results suggest that these interconnected signaling intermediates and receptor-based genes create a robust network to fine-tune the rice defense mechanisms in response to MOR infection.

## 4. Materials and Methods

### 4.1. Retrieving and Processing of High-Throughput Sequencing Data

#### 4.1.1. Retrieving and Processing of RNA-Seq Datasets

RNA-seq raw data from rice infected by MOR across different time courses were retrieved from the NCBI sequence read archive (SRA) database (https://www.ncbi.nlm.nih.gov/sra, accessed on 1 March 2024) using the NCBI SRA toolkit. A total of 201 RNA-seq samples were obtained at various infection stages, encompassing nine time points (8, 12, 24, 36, 48, 72, 96, 120, and 144 h post-infection, hpi) (Supplementary Data S1). SRA files were converted to Fastq format, followed by quality assessment with FastQC. Low-quality reads and adapter sequences were removed using the Trimmomatic v0.39 tool (Babraham Institute, UK, available at http://www.usadellab.org/cms/index.php?page=trimmomatic, accessed on 1 March 2024). Cleaned reads were mapped to the rice reference genome (IRGSP-1.0) using the HISAT2 tool (Center for Computational Biology, Johns Hopkins University, Baltimore, MD, USA), and BAM files were generated [69,70]. Gene counts were obtained using FeatureCounts and Samtools for downstream analysis [71].

#### 4.1.2. Retrieving and Processing of Microarray Datasets

Gene expression profiles of rice microarray transcriptomic datasets were retrieved from the GEO database within the NCBI platform. Rice microarray samples (n = 221) of MOR-infected and uninfected controls were retrieved, covering various expression profiles (Supplementary Data S1). All the microarray samples (.CEL) are deposited under two Affymetrix platforms, GPL6864 and GPL2025. The GEOquery package was utilized to import GEO data into the R programming environment using the accession codes (GSE) associated with the expression profiles of each sample series [72]. The Bioconductor package SimpleAffy (Fred Hutchinson Cancer Research Center, Seattle, WA, USA, available at: https://www.bioconductor.org/packages//2.7/bioc/html/simpleaffy.html, accessed on 1 March 2024).was also utilized for quality assessment. Poor-quality samples were discarded, and all the rest were used to analyze the differentially expressed genes [73]. Prop IDs were annotated to gene IDs based on the Affymetrix Rice Genome Array (GPL2025 and GPL6864).

### 4.2. Exploratory Data Analysis and Differential Gene Expression Analysis

In this study, we applied linear modeling integrated with empirical Bayes moderation (eBayes) for differential gene expression (DGE) analysis across both approaches, enhancing accuracy and reducing variability between RNA-seq and microarray datasets. Microarray dataset profiles were normalized with log_2_ transformation when the 99th percentile (quantile 0.99) exceeds 100, or if the 25th percentile (quantile 0.25) is greater than 0 and the range between the maximum and minimum values (quantile 1–quantile 0) exceeds 50. For RNA-seq datasets, data were normalized to counts per million (CPM) using the edgeR package (v.3.64.0) (Fred Hutchinson Cancer Research Center, Seasttle, WA, USA) in R programming software [30,31,74]. The stats R package (V.4.6.0) (R Foundation for Statistical Computing, Vienna, Austria) was used to generate the density distributions of each experimental condition (samples), the correlation matrix, and principal component analysis (PCA). Empirical Bayes batch correction was applied to the samples exhibiting batch effects to achieve a normal distribution across experimental groups, using the “limma precision weights” function. The Limma R package (Walter and Eliza Hall Institute of Medical Research, Melbourne, Victoria, Australia, available at; https://bioconductor.org/packages/release/bioc/html/limma.html, accessed on 1 March 2024).was used to identify DEGs between MOR-infected and healthy groups based on the programming language workflow outlined by GEO2R [72]. For RNA-seq data, edgeR and Limma packages were used to identify the DEGs between samples in each experiment based on the edgeR–Limma bioinformatic pipeline for RNA-seq analysis [31,75]. Significantly differentially expressed genes were screened using an adj. *p*-value ≤ 0.05 and log_2_FC ≥ 1. Furthermore, the robust rank aggregation (RRA) algorithm package in R software was used to integrate multiple DEG lists of individual experiments at both the RNA-seq and microarray datasets [76]. DEG lists were separately integrated for RNA-seq and microarray data at each time point, and final lists were arranged by top-regulated genes. The RRA algorithm assessed the ranking of DEGs in each experiment and compared them to the baseline across all the DEG lists.

### 4.3. Analysis of Receptor-Based Genes and Signaling Intermediate Genes in Rice Response to MOR Infection

Genes related to host–pathogen interactions, including extracellular receptor proteins and downstream signaling intermediates, were monitored among MOR-infected and mock rice samples. Members of extracellular and cytoplasmic receptors (RLKs, RLCKs, and WAKs) and downstream factors (MAPK and Ca^2+^ signaling genes) were all retrieved based on the International Rice Genome Sequencing Project/Rice Annotation Project Database (RAP-DB). A total of 385 RLCKs, 151 RLKs, and 105 WAKs were retrieved (Supplementary Data S2). A total of 31 MAPKs and 83 Ca^2+^-sensing encoding genes were determined (Supplementary Data S3). The retrieved gene IDs were then overlapped with all the DGEs of our current study to screen the upregulated and downregulated genes upon MOR infection across all transcriptomes.

### 4.4. PPI Network Analyses

PPI network of the identified key modules was constructed based on the STRING-functional gene interacting networks database (http://string-db.org, accessed on 1 March 2024) [77]. Both known and predicted PPI networks were screened. PPI matrices were conducted for DEGs of extracellular/cytoplasmic receptors and signaling intermediate genes. Connected nodes (genes) were directly imported into the Cytoscape tool (version 3.7.2) for visual editing of the PPI networks [78,79].

### 4.5. Statistical Analysis

Statistical pairs analysis between MOR-infected and healthy groups was compared based on Student’s *t*-tests (*p* ≤ 0.05) during the differential gene expression analysis. The correlation between samples was evaluated based on Pearson correlation coefficients.

## 5. Conclusions

Our study revealed that pattern-triggered immunity (PTI) machinery, including several receptor-based genes and signaling intermediate genes, concomitantly responds to MOR infection in rice plants, indicating that PTI machinery plays a crucial role in regulating rice immunity against MOR-caused blast disease. This research provides a comprehensive overview of the transcript dynamics of receptor-based genes under MOR stress, in which a total of 31 reporter-based genes, including receptor-like cytoplasmic kinases (RLCKs), wall-associated kinases (WAKs), and receptor-like kinases (RLKs), were commonly found among all transcriptomic data. This research facilitates a deeper molecular understanding of rice immunity regulation in response to MOR infection. Surface receptor and cytoplasmic receptor genes have highly dynamic transcript expression patterns in response to MOR infection and steadily increase upon infection progression. The majority of RLCKs, WAKs, and RLKs are largely activated rather than repressed across all the transcriptomic profiles. However, molecular functions and dynamic interactions among these mechanisms still warrant various further research questions to investigate the interplay possibilities between receptor-based genes and signaling intermediates in rice innate immunity against blast disease.

## Figures and Tables

**Table 1:** Differentially expressed gene (DEG) profiles of induced and repressed receptor-based genes and downstream signaling intermediate (Ca^2+^ and MAPKs) genes derived from the RNA-seq and microarray datasets.

Pathogen Infection Time Course (HPI)	Total DEGs		Extracellular Receptor Genes		Signaling-MAPK/Ca^2+^ Signaling Genes		*N*—Samples	Expression Profile (GEO-Code)	Data Type
	Up	Down	Up	Down	Up	Down			
12 h (T1)	785	386	19	3	3	2	3 paired	GSE95394	arrays
24 h (T1)	2910	2512	66	13	13	6	3 paired	GSE95394	arrays
48 h (T2)	3151	2059	72	32	24	11	3 paired	GSE95394	arrays
72 h (T3)	4425	6352	83	50	24	13	3 paired	GSE95394	arrays
24 h (T1)	511	791	13	12	2	5	12 MOR	GSE30941	arrays
24 h (T1)	1933	975	67	1	11	3	9 paired	GSE41798	arrays
72 h (T3)	2535	2862	28	23	5	7	2 paired	GSE28308	arrays
48 h (T2)	2875	1913	92	11	25	0	2 paired	GSE18361 *	arrays
96 h (T4)	1500	1324	71	9	12	0	2 paired	GSE18361 *	arrays
144 h (T4)	1182	720	50	3	9	0	2 paired	GSE18361 *	arrays
24 h (T1)	3316	509	112	6	27	0	6 paired	GSE62894	arrays
48 h (T2)	5905	1721	108	26	44	1	6 paired	GSE62894	arrays
72 h (T3)	3568	1655	146	63	46	9	6 paired	GSE62894	arrays
120 h (T4)	5427	7804	130	98	29	18	6 paired	GSE62894	arrays
24 h (T1)	2911	175	111	6	23	0	6 paired	GSE62893	arrays
48 h (T2)	4827	1962	98	25	43	1	6 paired	GSE62893	arrays
72 h (T3)	3568	1655	84	31	24	2	6 paired	GSE62893	arrays
120 h (T4)	2329	2372	52	26	16	15	6 paired	GSE62893	arrays
24 h (T1)	1697	1748	14	13	2	0	6 paired	GSE62895 *	arrays
48 h (T2)	1247	1855	17	23	3	0	6 paired	GSE62895 *	arrays
72 h (T3)	1412	1680	14	19	4	0	6 paired	GSE62895 *	arrays
120 h (T4)	1523	1364	16	13	4	0	6 paired	GSE62895 *	arrays
8 h (T1)	1027	1760	33	25	14	9	9 MOR	PRJEB45007	RNA-seq
16 h (T1)	1868	2485	31	23	10	4	9 MOR	PRJEB45007	RNA-seq
24 h (T1)	2162	1859	52	30	15	2	9 MOR	PRJEB45007	RNA-seq
48 h (T2)	2942	1487	89	21	22	3	9 MOR	PRJEB45007	RNA-seq
72 h (T3)	4337	3294	145	45	35	6	9 MOR	PRJEB45007	RNA-seq
96 h (T4)	4902	3807	156	47	34	4	9 MOR	PRJEB45007	RNA-seq
144 h (T4)	4315	3604	129	45	26	5	9 MOR	PRJEB45007	RNA-seq
12 h (T1)	2243	1913	37	24	5	7	3 paired	PRJNA545418	RNA-seq
24 h (T1)	2375	1951	38	27	10	8	3 paired	PRJNA545418	RNA-seq
12 h (T1)	1749	1965	27	22	5	6	3 paired	PRJNA545418	RNA-seq
24 h (T1)	1734	2130	24	18	7	8	3 paired	PRJNA545418	RNA-seq
12 h (T1)	3098	1869	66	27	18	0	6 paired	PRJNA661210	RNA-seq
24 h (T1)	1871	1169	76	10	11	0	6 paired	PRJNA661210	RNA-seq
36 h (T2)	2497	1921	95	15	16	4	6 paired	PRJNA661210	RNA-seq
48 h (T2)	2291	2011	84	11	14	2	6 paired	PRJNA661210	RNA-seq
24 h (T1)	719	668	47	9	6	2	3 paired	PRJNA1062412	RNA-seq
48 h (T2)	946	803	62	3	6	2	3 paired	PRJNA1062412	RNA-seq
72 h (T3)	719	298	54	6	5	0	3 paired	PRJNA1062412	RNA-seq
24 h (T1)	1560	945	35	15	11	6	2 MOR	PRJNA590671	RNA-seq
72 h (T3)	2181	1056	29	23	13	3	2 MOR	PRJNA590671	RNA-seq
120 (T4)	2046	1358	41	19	15	8	2 MOR	PRJNA590671	RNA-seq
24 h (T1)	1683	1412	48	11	8	3	2 MOR	PRJNA310071	RNA-seq
48 h (T2)	1965	1684	35	13	14	3	2 MOR	PRJNA310071	RNA-seq
48 h (T2)	834	610	20	18	5	4	2 paired	PRJNA563035 *	RNA-seq
72 h (T3)	1120	918	13	10	2	5	2 paired	PRJNA563035 *	RNA-seq
96 h (T4)	1023	457	30	11	3	5	2 paired	PRJNA563035 *	RNA-seq
24 h (T1)	2991	3014	37	40	10	17	6 paired	PRJNA634330	RNA-seq

* GSE18361 and * GSE62895: root samples * PRJNA563035: spike samples; *N*—samples: (251 MOR-infected samples/171 mock–healthy samples across all time points, including zero-time infection); Total DEGs: represent the transcriptional profiles of whole differentially expressed genes responsive to MOR infection in each experiment. Differentially expressed genes (DEGs), including total DEGs of expression profiles, up and downregulated genes of receptor-based genes, and downstream signaling genes, are all extracted from the analyzed dataset. The cutoff for DEG analysis is *p* ≤ 0.05, with log_2_FC = 1, (−1). Where “Up” represents “upregulated/induced genes” and “Down” represents “downregulated/repressed genes”. Extracellular receptor genes include all receptor-like kinase genes (*RLKs*, *RLCKs*, *WAKs*). HPI: hours post-infection. GEO: Gene Expression Omnibus-NCBI database. MOR: *Magnaporthe oryzae.* T1:8–24 h post-inoculation; T2: 36–48 h post-inoculation; T3: 72 h post-inoculation; T4: 96–144 h post-inoculation.

**Table 2:** Top extracellular and cytoplasmic receptors most frequently upregulated genes in response to MOR infection in rice transcriptomic datasets.

Gene ID	Gene Name	Receptor Type	Frequency Across the Analyzed Datasets			
			Infection Stage	No. of Samples		
				Total	Arrays	RNA-Seq
Os08G0457400	*OsRLCK255*	Cytoplasmic-like kinase	T1-T2-T3-T4	190	72	118
Os02G0807900	*OsWAK21*	WALL-associated kinase	T1	21	18	3
Os02G0807200	*OsWAK18*	WALL-associated kinase	T1-T2-T3-T4	81	72	9
Os09G0561500	*WAK90*	WALL-associated kinase	T1-T3-T4	90	54	36
Os04G0226600	*OsRLCK138*	Cytoplasmic-like kinase	T1-T3-T4	45	18	27
Os11G0666200	*OsRLCK345*	Cytoplasmic-like kinase	T1-T2-T3-T4	72	72	0
Os02G0632800	*OsWAK14*	WALL-associated kinase	T1-T2-T3	54	54	0
Os07G0541700	*OsRLCK237*	Cytoplasmic-like kinase	T1-T2-T3-T4	72	72	0
Os02G0807800	*OsWAK20*	WALL-associated kinase	T1-T2-T3-T4	126	72	54
Os07G0686800	*OsRLCK241*	Cytoplasmic-like kinase	T1–T2	63	36	27
Os11G0557500	*OsLysM-RLK7*	Lysin-motif extracellular receptor protein	T1-T2-T3	54	54	0
Os11G0514500	*--*	Leucine repeat with extracellular domain	T2-T4	36	36	0
Os08G0374600	*OsRLCK253*	Cytoplasmic-like kinase	T2-T3-T4	54	54	0
Os12G0486900	*OsRLCK369*	Cytoplasmic-like kinase	T2-T3-T4	63	54	9
Os07G0534500	*OsRLCK233*	Cytoplasmic-like kinase	T2	27	18	9
Os03G0407900	*OsRLCK110*	Cytoplasmic-like kinase	T2–T3	81	36	45
Os04G0631800	*OsRLCK162*	Cytoplasmic-like kinase	T2–T3	36	36	0
Os02G0193000	*OsLysM-RLK1*	Lysin-motif-extracellular receptor	T3	18	18	0
Os02G0811200	*OsWAK24*	WALL-associated kinase	T1-T2-T3-T4	158	36	122
Os04G0598900	*OsWAK50*	WALL-associated kinase	T3–T4	45	18	27
Os01G0136400	*OsWAK1*	WALL-associated kinase	T1–T4	40	18	22
Os04G0127500	*OsWAK29*	WALL-associated kinase	T2-T3-T4	57	54	3
Os03G0264300	*OsRLCK106*	Cytoplasmic-like kinase	T4	44	26	18
OS02G0553000	*LRR-RLK*	Leucine-rich repeat receptor-like kinase	T1-T3-T4	60	0	60

T1: 8–24 h post-inoculation; T2: 36–48 h post-inoculation; T3: 72 h post-inoculation; T4: 96–144 h post-inoculation.

**Table 3:** Unique extracellular and cytoplasmic receptor-based genes are frequently expressed under MOR infection at different specific infection stages in the rice transcriptomic datasets.

Gene ID	Gene Name	Receptor Type	Stage	Regulation
Os04G0369100	*OsRLCK145*	Cytoplasmic-receptor-like kinase	T1	Up
Os01G0137200	*OsRLCK20*	Cytoplasmic-receptor-like kinase	T1	Up
Os11G0225000	*OsRLCK319*	Cytoplasmic-receptor-like kinase	T1	Up
Os06G0541600	*OsRLCK206*	Cytoplasmic-receptor-like kinase	T1	Up
Os04G0369000	*OsRLCK144*	Cytoplasmic-receptor-like kinase	T1	Up
Os03G0241600	*OsRLCK105*	Cytoplasmic-receptor-like kinase	T1	Up
Os03G0179400	*OsRLCK103*	Cytoplasmic-receptor-like kinase	T1	Up
Os02G0186500	*OsRLCK64*	Cytoplasmic-receptor-like kinase	T1	Up
Os01G0929200	*OsRLCK53*	Cytoplasmic-receptor-like kinase	T1	Up
Os01G0114900	*OsRLCK9*	Cytoplasmic-receptor-like kinase	T1	Up
Os11G0609500	*OsRLCK339*	Cytoplasmic-receptor-like kinase	T1	Up
Os01G0545500	*OsRLCK36*	Cytoplasmic-receptor-like kinase	T1	Up
Os06G0727400	*OsRLCK220*	Cytoplasmic-receptor-like kinase	T1	Up
Os01G0117300	*OsRLCK17*	Cytoplasmic-receptor-like kinase	T1	Up
Os01G0784500	*OsRLCK44*	Cytoplasmic-receptor-like kinase	T1	Up
Os01G0117200	*OsRLCK16*	Cytoplasmic-receptor-like kinase	T1	Up
Os04G0365100	*OsWAK37*	WALL-associated kinase	T1	Up
Os11G0694100	*OsWAK123*	WALL-associated kinase	T1	Up
Os05G0463000	*OsRLCK188*	Cytoplasmic-receptor-like kinase	T2	Up
Os06G0663900	*OsRLCK212*	Cytoplasmic-receptor-like kinase	T2	Up
Os03G0825800	*OsRLCK120*	Cytoplasmic-receptor-like kinase	T2	Up
Os01G0689900	*OsWAK10*	WALL-associated kinase	T2	Up
Os02G0227700	*OsRLK5*	Extracellular receptor	T3	Up
Os06G0663200	*OsRLCK211*	Cytoplasmic-receptor-like kinase	T3	Up
Os02G0787200	*OsRLCK87*	Cytoplasmic-receptor-like kinase	T3	Up
Os01G0789200	*OsRLCK45*	Cytoplasmic-receptor-like kinase	T3	Up
Os06G0202900	*OsRLCK203*	Cytoplasmic-receptor-like kinase	T3	Up
Os03G0283900	*OsRLCK108*	Cytoplasmic-receptor-like kinase	T3	Up
Os12G0615100	*OsWAK128*	WALL-associated kinase	T3	Up
Os11G0549300	*OsLysM-RLK8*	Lysin-motif extracellular receptor	T4	Up
Os04G0655400	*OsRLCK169*	Cytoplasmic-receptor-like kinase	T4	Up
Os09G0479200	*OsRLCK275*	Cytoplasmic-receptor-like kinase	T4	Up
Os01G0267800	*OsRLCK29*	Cytoplasmic-receptor-like kinase	T4	Up
Os02G0639100	*OsRLCK78*	Cytoplasmic-receptor-like kinase	T4	Up
Os02G0565500	*OsRLCK74*	Cytoplasmic-receptor-like kinase	T4	Up
Os09G0533600	*OsRLCK278*	Cytoplasmic-receptor-like kinase	T4	Up
Os07G0537200	*OsRLCK234*	Cytoplasmic-receptor-like kinase	T4	Up
Os04G0517700	*OsWAK51*	WALL-associated kinase	T4	Up
Os03G0841100	*OsWAK28*	WALL-associated kinase	T4	Up
Os01G0137500	*OsRLCK22*	Cytoplasmic-receptor-like kinase	T1	Down
Os01G0546000	*OsLysM-RLK3*	Lysin-motif extracellular receptor	T2	Down
Os08G0506400	*OsRLCK257*	Cytoplasmic-receptor-like kinase	T2	Down
Os04G0220300	*OsWAK30*	WALL-associated kinase	T2	Down
Os10G0200000	*OsRLCK295*	Cytoplasmic-receptor-like kinase	T3	Down
Os11G0300700	*OsRLCK325*	Cytoplasmic-receptor-like kinase	T3	Down
Os04G0654600	*OsRLCK167*	Cytoplasmic-receptor-like kinase	T3	Down
Os01G0929200	*OsRLCK53*	Cytoplasmic-receptor-like kinase	T4	Down
Os10G0431900	*OsRLCK300*	Cytoplasmic-receptor-like kinase	T4	Down
Os11G0445300	*OsRLCK327*	Cytoplasmic-receptor-like kinase	T4	Down
Os01G0114600	*OsRLCK8*	Cytoplasmic-receptor-like kinase	T4	Down
Os01G0117400	*OsRLCK18*	Cytoplasmic-receptor-like kinase	T4	Down
Os05G0100700	*OsRLCK175*	Cytoplasmic-receptor-like kinase	T4	Down
Os01G0296000	*OsRLCK30*	Cytoplasmic-receptor-like kinase	T4	Down
Os01G0117000	*OsRLCK15*	Cytoplasmic-receptor-like kinase	T4	Down
Os03G0844100	*OsRLCK123*	Cytoplasmic-receptor-like kinase	T4	Down
Os02G0152300	*OsRLCK61*	Cytoplasmic-receptor-like kinase	T4	Down
Os01G0117300	*OsRLCK17*	Cytoplasmic-receptor-like kinase	T4	Down
Os04G0619600	*OsRLCK161*	Cytoplasmic-receptor-like kinase	T4	Down
Os07G0134200	*OsRLCK222*	Cytoplasmic-receptor-like kinase	T4	Down
Os03G0274800	*OsRLCK107*	Cytoplasmic-receptor-like kinase	T4	Down
Os03G0130900	*OsRLCK96*	Cytoplasmic-receptor-like kinase	T4	Down
Os06G0168800	*OsRLCK200*	Cytoplasmic-receptor-like kinase	T4	Down
Os08G0200500	*OsRLCK247*	Cytoplasmic-receptor-like kinase	T4	Down
Os11G0194900	*OsRLCK315*	Cytoplasmic-receptor-like kinase	T4	Down
Os01G0115600	*OsRLCK11*	Cytoplasmic-receptor-like kinase	T4	Down
Os01G0310500	*OsRLCK31*	Cytoplasmic-receptor-like kinase	T4	Down
Os10G0180800	*WAK112*	WALL-associated kinase	T4	Down
Os09G0471400	*OsWAK81*	WALL-associated kinase	T4	Down
Os04G0365100	*OsWAK37*	WALL-associated kinase	T4	Down
Os09G0471800	*OsWAK85*	WALL-associated kinase	T4	Down
Os04G0286300	*OsWAK33*	WALL-associated kinase	T4	Down
Os12G0615300	*OsWAK129*	WALL-associated kinase	T4	Down

T1: 8–24 h post-inoculation; T2: 36–48 h post-inoculation; T3: 72 h post-inoculation; T4: 96–144 h post-inoculation; “Up” represents “upregulated/induced genes” while “Down” represents “downregulated/repressed genes”.

**Table 4:** Top downstream signaling genes (Ca^2+^/MAPK) that are most frequently upregulated in response to MOR infection in rice transcriptomic datasets.

Gene ID	Gene Name	Protein Domain	Frequency Across the Analyzed Datasets			
			Infection Stage	No. of Samples		
				Total	Arrays	RNA-Seq
Os01G0955100	*OsCML31*	Calmodulin-like protein	T1-T2-T3-T4	176	54	122
Os12G0603800	*OsCML5*	Calmodulin-like protein	T1-T2-T3-T4	131	18	113
Os05G0577500	*OsCML14*	Calmodulin-like protein	T1–T4	63	36	27
Os11G0105000	*OsCML25*	Calmodulin-like protein	T3–T4	60	18	42
Os12G0104900	*OsCML26*	Calmodulin-like protein	T4	27	18	9
Os10G0418100	*ACA8*	Ca^2+^ P-type ATPase	T1-T2-T3-T4	45	36	9
Os04G0644900	*OsNTMC2T2.1*	N-terminal trans-membrane	T1	18	18	0
Os03G0397400	*OsCAX2*	Vacuolar cation exchanger protein	T1–T3	54	21	33
Os12G0624200	*OsCCX4*	Ca^2+^ exchanger	T3–T4	36	36	0
Os04G0584600	*OsCDPK13*	Ca^2+^-dependent protein kinase	T1–T3	60	18	42
Os07G0631700	*EF-hand*	EF-hand type domain	T3	54	54	0
Os07G0584100	*OsWNK5*	MAP kinase-like protein	T1-T2-T3	81	72	9
Os12G0162100	*OsWNK9*	MAP kinase-like protein	T3	18	18	0
Os03G0415200	*MAP3K*	MAP kinase-like protein	T1-T2-T3	117	72	45
Os01G0699500	*MAP3K6*	MAP kinase-like protein	T2-T3-T4	54	54	0
Os06G0147800	*OsMKK1*	MAP (2) K	T1-T2-T4	120	72	48
Os01G0510100	*OsMKK6*	MAP (2) K	T2–T3	36	36	0
Os03G0285800	*OsMSRMK2*	MAPK	T1-T2-T3-T4	176	54	122
Os10G0533600	*OsMPK6*	MAPK	T1-T2-T3	72	36	36
Os02G0135200	*OsMPK13*	MAPK	T2–T4	63	36	27

T1: 8–24 h post-inoculation; T2: 36–48 h post-inoculation; T3: 72 h post-inoculation; T4: 96–144 h post-inoculation.

## Data Availability

The datasets analyzed in this meta-analysis during the current study are all available in the NCBI datasets. The microarray expression profiles are available in NCBI’s Gene Expression Omnibus (GEO) under GEO accessions: GSE95394, GSE30941, GSE41798, GSE28308, GSE18361, GSE62894, GSE62893, and GSE62895. For RNA seq datasets, all datasets are available under eight Bio-Projects accessions: PRJEB45007, PRJNA545418, PRJNA661210, PRJNA1062412, PRJNA590671, PRJNA563035, PRJNA310071, and PRJNA634330. Furthermore, the extra-related output findings of this research are deposited in 16 additional Supplementary Files, including 12 Supplementary Data Files and Figures S1–S4. Any further inquiries can be directed to the corresponding authors.

## Supplementary Materials

The following are available online at https://www.mdpi.com/article/10.3390/ijms26104618/s1.

## Abbreviations

The following abbreviations are used in this manuscript:

MOR	*Magnaporthe oryzae*
RNA-seq	RNA sequencing
GEO	Gene Expression Omnibus
GSE	Gene Expression Omnibus series
GPL	Gene expression platform
CPM	Counts per million
PCA	Principal component analysis
DEGs	Differentially expressed genes
PPIs	Protein–protein interactions
PAMPs	pathogen-associated molecular patterns
DAMPs	host damage-associated molecular patterns
PTI	Pathogen-triggered immunity
WAK	Wall-associated kinase
RLK	Receptor-like kinases
RLCK	Receptor-like cytoplasmic kinase
MAPK (MPK	Mitogen-activated protein kinases
MAPKK (MKK)	MAPK kinases
MAPKKK	MAPK kinases kinase
ROS	Reactive oxygen species
NLR	nucleotide binding and leucine-rich-repeat

## References

[1] Asibi A.E., Chai Q., Coulter J.A. (2019). Rice blast: A disease with implications for global food security. Agronomy.

[2] Younas M.U., Ahmad I., Qasim M., Ijaz Z., Rajput N., Memon S.P., Zaman W.U., Jiang X., Zhang Y., Zuo S. (2024). Progress in the management of rice blast disease: The role of avirulence and resistance genes through gene-for-gene interactions. Agronomy.

[3] Kou Y., Shi H., Qiu J., Tao Z., Wang W. (2024). Effectors and environment modulating rice blast disease: From understanding to effective control. Trends Microbiol..

[4] Yuan M., Ngou B.P.M., Ding P., Xin X.F. (2021). PTI-ETI crosstalk: An integrative view of plant immunity. Curr. Opin. Plant Biol..

[5] Ngou B.P.M., Ahn H.K., Ding P., Jones J.D.G. (2021). Mutual potentiation of plant immunity by cell-surface and intracellular receptors. Nature.

[6] Yuan M., Jiang Z., Bi G., Nomura K., Liu M., Wang Y., Cai B., Zhou J.M., He S.Y., Xin X.F. (2021). Pattern-recognition receptors are required for NLR-mediated plant immunity. Nature.

[7] Lin L., Wu J., Jiang M., Wang Y. (2021). Plant mitogen-activated protein kinase cascades in environmental stresses. Int. J. Mol. Sci..

[8] Bhar A., Chakraborty A., Roy A. (2023). The captivating role of calcium in plant-microbe interaction. Front. Plant Sci..

[9] Edel K.H., Marchadier E., Brownlee C., Kudla J., Hetherington A.M. (2017). The Evolution of Calcium-Based Signalling in Plants. Curr. Biol..

[10] Liu W., Wang G.-L. (2016). Plant innate immunity in rice: A defense against pathogen infection. Natl. Sci. Rev..

[11] Ngou B.P.M., Jones J.D.G., Ding P. (2022). Plant immune networks. Trends Plant Sci..

[12] Wang C., Luan S. (2024). Calcium homeostasis and signaling in plant immunity. Curr. Opin. Plant Biol..

[13] Li B., Meng X., Shan L., He P. (2016). Transcriptional Regulation of Pattern-Triggered Immunity in Plants. Cell Host Microbe.

[14] Choi H.W., Klessig D.F. (2016). DAMPs, MAMPs, and NAMPs in plant innate immunity. BMC Plant Biol..

[15] Ding L.N., Li Y.T., Wu Y.Z., Li T., Geng R., Cao J., Zhang W., Tan X.L. (2022). Plant disease resistance-related signaling pathways: Recent progress and future prospects. Int. J. Mol. Sci..

[16] He Y., Zhou J., Shan L., Meng X. (2018). Plant cell surface receptor-mediated signaling—A common theme amid diversity. J. Cell Sci..

[17] Ngou B.P.M., Wyler M., Schmid M.W., Kadota Y., Shirasu K. (2024). Evolutionary trajectory of pattern recognition receptors in plants. Nat. Commun..

[18] Liu X., Wang Z., Tian Y., Zhang S., Li D., Dong W., Zhang C., Zhang Z. (2021). Characterization of wall-associated kinase/wall-associated kinase-like (WAK/WAKL) family in rose (*Rosa chinensis*) reveals the role of RcWAK4 in Botrytis resistance. BMC Plant Biol..

[19] Kawano Y., Shimamoto K. (2013). Early signaling network in rice PRR-mediated and R-mediated immunity. Curr. Opin. Plant Biol..

[20] Jalilian A., Bagheri A., Chalvon V., Meusnier I., Kroj T., Kakhki A.M. (2023). The RLCK subfamily VII-4 controls pattern-triggered immunity and basal resistance to bacterial and fungal pathogens in rice. Plant J..

[21] Brutus A., Sicilia F., Macone A., Cervone F., De Lorenzo G. (2010). A domain swap approach reveals a role of the plant wall-associated kinase 1 (WAK1) as a receptor of oligogalacturonides. Proc. Natl. Acad. Sci. USA.

[22] Kohorn B.D., Kohorn S.L., Saba N.J., Martinez V.M. (2014). Requirement for pectin methyl esterase and preference for fragmented over native pectins for wall-associated kinase-activated, EDS1/PAD4-dependent stress response in *Arabidopsis*. J. Biol. Chem..

[23] Ma H., Chen J., Zhang Z., Ma L., Yang Z., Zhang Q., Li X., Xiao J., Wang S. (2017). MAPK kinase 10.2 promotes disease resistance and drought tolerance by activating different MAPKs in rice. Plant J..

[24] Chen J., Wang L., Yuan M. (2021). Update on the roles of rice MAPK cascades. Int. J. Mol. Sci..

[25] Zhang J., Coaker G., Zhou J.M., Dong X. (2020). Plant immune mechanisms: From reductionistic to holistic points of View. Mol. Plant.

[26] Lodha T.D., Basak J. (2012). Plant-pathogen interactions: What microarray tells about it?. Mol. Biotechnol..

[27] Devanna B.N., Jain P., Solanke A.U., Das A., Thakur S., Singh P.K., Kumari M., Dubey H., Jaswal R., Pawar D. (2022). Understanding the dynamics of blast resistance in rice-*Magnaporthe oryzae* interactions. J. Fungi.

[28] Sharma T.R., Rai A.K., Gupta S.K., Vijayan J., Devanna B.N., Ray S. (2012). Rice Blast Management Through Host-Plant Resistance: Retrospect and Prospects. Agric. Res..

[29] Ding L., Xu X., Kong W., Xia X., Zhang S., Liu L.-W., Liu A., Zou L. (2020). Genome-wide identification and expression analysis of rice *NLR* genes responsive to the infections of *Xanthomonas oryzae* pv. *oryzae* and *Magnaporthe oryzae*. Physiol. Mol. Plant Pathol..

[30] Ritchie M.E., Phipson B., Wu D., Hu Y., Law C.W., Shi W., Smyth G.K. (2015). Limma powers differential expression analyses for RNA-sequencing and microarray studies. Nucleic Acids Res..

[31] Law C.W., Alhamdoosh M., Su S., Smyth G.K., Ritchie M.E. (2016). RNA-seq analysis is easy as 1-2-3 with limma, Glimma and edgeR. F1000Research.

[32] Jolliffe I.T., Cadima J. (2016). Principal component analysis: A review and recent developments. Philos. Trans. A Math. Phys. Eng. Sci..

[33] Wei T., Ou B., Li J., Zhao Y., Guo D., Zhu Y., Chen Z., Gu H., Li C., Qin G. (2013). Transcriptional profiling of rice early response to *Magnaporthe oryzae* identified OsWRKYs as important regulators in rice blast resistance. PLoS ONE.

[34] Wang Y., Kwon S.J., Wu J., Choi J., Lee Y.H., Agrawal G.K., Tamogami S., Rakwal R., Park S.R., Kim B.G. (2014). Transcriptome Analysis of Early Responsive Genes in Rice during *Magnaporthe oryzae* Infection. Plant Pathol. J..

[35] Mosquera G., Giraldo M.C., Khang C.H., Coughlan S., Valent B. (2009). Interaction transcriptome analysis identifies *Magnaporthe oryzae* BAS1-4 as Biotrophy-associated secreted proteins in rice blast disease. Plant Cell.

[36] Singh V., Sharma V., Katara P. (2016). Comparative transcriptomics of rice and exploitation of target genes for blast infection. Agri Gene.

[37] Wang P., Yao S., Kosami K.I., Guo T., Li J., Zhang Y., Fukao Y., Kaneko-Kawano T., Zhang H., She Y.M. (2020). Identification of endogenous small peptides involved in rice immunity through transcriptomics- and proteomics-based screening. Plant Biotechnol. J..

[38] Abbruscato P., Nepusz T., Mizzi L., Del Corvo M., Morandini P., Fumasoni I., Michel C., Paccanaro A., Guiderdoni E., Schaffrath U. (2012). *OsWRKY22*, a monocot *WRKY* gene, plays a role in the resistance response to blast. Mol. Plant Pathol..

[39] Jain P., Singh P.K., Kapoor R., Khanna A., Solanke A.U., Krishnan S.G., Singh A.K., Sharma V., Sharma T.R. (2017). Understanding host-pathogen interactions with expression profiling of NILs carrying rice-blast resistance *Pi9* gene. Front. Plant Sci..

[40] Kumar V., Jain P., Venkadesan S., Karkute S.G., Bhati J., Abdin M.Z., Sevanthi A.M., Mishra D.C., Chaturvedi K.K., Rai A. (2021). Understanding Rice-*Magnaporthe Oryzae* Interaction in Resistant and Susceptible Cultivars of Rice under Panicle Blast Infection Using a Time-Course Transcriptome Analysis. Genes.

[41] Antony A., Veerappapillai S., Karuppasamy R. (2024). Deciphering early responsive signature genes in rice blast disease: An integrated temporal transcriptomic study. J. Appl. Genet..

[42] Kong W., Ding L., Xia X. (2020). Identification and characterization of genes frequently responsive to *Xanthomonas oryzae* pv. *oryzae* and *Magnaporthe oryzae* infections in rice. BMC Genom..

[43] Tian L., Shi S., Nasir F., Chang C., Li W., Tran L.P., Tian C. (2018). Comparative analysis of the root transcriptomes of cultivated and wild rice varieties in response to *Magnaporthe oryzae* infection revealed both common and species-specific pathogen responses. Rice.

[44] Yang, Dewei, Li S., Xiao Y., Lu L., Zheng Z., Tang D., Cui H. (2021). Transcriptome analysis of rice response to blast fungus identified core genes involved in immunity. Plant Cell Environ..

[45] Shu X., Wang A., Jiang B., Jiang Y., Xiang X., Yi X., Li S., Deng Q., Wang S., Zhu J. (2021). Genome-wide association study and transcriptome analysis discover new genes for bacterial leaf blight resistance in rice (*Oryza sativa* L.). BMC Plant Biol..

[46] Bakade R., Ingole K.D., Deshpande S., Pal G., Patil S.S., Bhattacharjee S., Prasannakumar M.K., Ramu V.S. (2021). Comparative Transcriptome Analysis of Rice Resistant and Susceptible Genotypes to *Xanthomonas oryzae* pv. *oryzae* Identifies Novel Genes to Control Bacterial Leaf Blight. Mol. Biotechnol..

[47] Sana T.R., Fischer S., Wohlgemuth G., Katrekar A., Jung K.H., Ronald P.C., Fiehn O. (2010). Metabolomic and transcriptomic analysis of the rice response to the bacterial blight pathogen *Xanthomonas oryzae* pv. *oryzae*. Metabolomics.

[48] Li Z., Shen S., Xia K., Zhang M., Zeng X. (2024). Integrative genomic and transcriptomic analysis of *Xanthomonas oryzae* pv. *oryzae* pathotype IV, V, and IX in China reveals rice defense-responsive genes. Phytopathol. Res..

[49] Chen P., Zhang X., Li X., Sun B., Yu H., Liu Q., Jiang L., Mao X., Zhang J., Lv S. (2024). Transcriptome Analysis of Rice Near-Isogenic Lines Inoculated with Two Strains of *Xanthomonas oryzae* pv. *oryzae*, AH28 and PXO99(A). Plants.

[50] Iqbal O., Yang X., Wang Z., Li D., Wen J., Ding J., Wang C., Li C., Wang Y. (2025). Comparative transcriptome and genome analysis between susceptible Zhefang rice variety Diantun 502 and its resistance variety Diantun 506 upon *Magnaporthe oryzae* infection. BMC Plant Biol..

[51] Du Y., Liang D., Qi Z., Yu J., Zhang R., Song T., Yu M., Cao H., Pan X., Wang S. (2024). Transcriptome and differential expression analysis revealed the pathogenic-related genes in *Magnaporthe oryzae* during leaf and panicle infection. Phytopathol. Res..

[52] Xiao X., Wang R., Guo W., Khaskhali S., Fan R., Zhao R., Li C., He C., Niu X., Chen Y. (2022). The receptor-like cytoplasmic kinase *OsRLCK118* regulates plant development and basal immunity in rice (*Oryza sativa* L.). Trop. Plants.

[53] Wang J., Liu X., Zhang A., Ren Y., Wu F., Wang G., Xu Y., Lei C., Zhu S., Pan T. (2019). A cyclic nucleotide-gated channel mediates cytoplasmic calcium elevation and disease resistance in rice. Cell Res..

[54] Yamaguchi K., Yamada K., Ishikawa K., Yoshimura S., Hayashi N., Uchihashi K., Ishihama N., Kishi-Kaboshi M., Takahashi A., Tsuge S. (2013). A receptor-like cytoplasmic kinase targeted by a plant pathogen effector is directly phosphorylated by the chitin receptor and mediates rice immunity. Cell Host Microbe.

[55] Boller T., He S.Y. (2009). Innate Immunity in Plants: An Arms Race Between Pattern Recognition Receptors in Plants and Effectors in Microbial Pathogens. Science.

[56] Boller T., Felix G. (2009). A renaissance of elicitors: Perception of microbe-associated molecular patterns and danger signals by pattern-recognition receptors. Annu. Rev. Plant Biol..

[57] Delteil A., Gobbato E., Cayrol B., Estevan J., Michel-Romiti C., Dievart A., Kroj T., Morel J.B. (2016). Several wall-associated kinases participate positively and negatively in basal defense against rice blast fungus. BMC Plant Biol..

[58] Rui Y., Dinneny J.R. (2020). A wall with integrity: Surveillance and maintenance of the plant cell wall under stress. New Phytol..

[59] Zhang N., Pombo M.A., Rosli H.G., Martin G.B. (2020). Tomato wall-associated kinase SlWak1 depends on Fls2/Fls3 to promote apoplastic immune responses to *Pseudomonas syringae*. Plant Physiol..

[60] Dmochowska-Boguta M., Kloc Y., Zielezinski A., Werecki P., Nadolska-Orczyk A., Karlowski W.M., Orczyk W. (2020). *TaWAK6* encoding wall-associated kinase is involved in wheat resistance to leaf rust similar to adult plant resistance. PLoS ONE.

[61] Saintenac C., Lee W.S., Cambon F., Rudd J.J., King R.C., Marande W., Powers S.J., Berges H., Phillips A.L., Uauy C. (2018). Wheat receptor-kinase-like protein Stb6 controls gene-for-gene resistance to fungal pathogen *Zymoseptoria tritici*. Nat. Genet..

[62] Zhong Z., Marcel T.C., Hartmann F.E., Ma X., Plissonneau C., Zala M., Ducasse A., Confais J., Compain J., Lapalu N. (2017). A small secreted protein in *Zymoseptoria tritici* is responsible for avirulence on wheat cultivars carrying the *Stb6* resistance gene. New Phytol..

[63] Jiang R., Zhou S., Da X., Yan P., Wang K., Xu J., Mo X. (2023). OsMKK6 Regulates Disease Resistance in Rice. Int. J. Mol. Sci..

[64] Ma H., Li J., Ma L., Wang P., Xue Y., Yin P., Xiao J., Wang S. (2021). Pathogen-inducible OsMPKK10.2-OsMPK6 cascade phosphorylates the Raf-like kinase OsEDR1 and inhibits its scaffold function to promote rice disease resistance. Mol. Plant.

[65] Ueno Y., Yoshida R., Kishi-Kaboshi M., Matsushita A., Jiang C.J., Goto S., Takahashi A., Hirochika H., Takatsuji H. (2015). Abiotic Stresses Antagonize the Rice Defence Pathway through the Tyrosine-Dephosphorylation of OsMPK6. PLoS Pathog..

[66] Kishi-Kaboshi M., Okada K., Kurimoto L., Murakami S., Umezawa T., Shibuya N., Yamane H., Miyao A., Takatsuji H., Takahashi A. (2010). A rice fungal MAMP-responsive MAPK cascade regulates metabolic flow to antimicrobial metabolite synthesis. Plant J..

[67] Wang G., Roux B., Feng F., Guy E., Li L., Li N., Zhang X., Lautier M., Jardinaud M.F., Chabannes M. (2015). The Decoy Substrate of a Pathogen Effector and a Pseudokinase Specify Pathogen-Induced Modified-Self Recognition and Immunity in Plants. Cell Host Microbe.

[68] Yamada K., Yamaguchi K., Shirakawa T., Nakagami H., Mine A., Ishikawa K., Fujiwara M., Narusaka M., Narusaka Y., Ichimura K. (2016). The *Arabidopsis* CERK1-associated kinase PBL27 connects chitin perception to MAPK activation. EMBO J..

[69] Kawahara Y., de la Bastide M., Hamilton J.P., Kanamori H., McCombie W.R., Ouyang S., Schwartz D.C., Tanaka T., Wu J., Zhou S. (2013). Improvement of the *Oryza sativa* Nipponbare reference genome using next generation sequence and optical map data. Rice.

[70] Kim D., Paggi J.M., Park C., Bennett C., Salzberg S.L. (2019). Graph-based genome alignment and genotyping with HISAT2 and HISAT-genotype. Nat. Biotechnol..

[71] Liao Y., Smyth G.K., Shi W. (2014). FeatureCounts: An efficient general purpose program for assigning sequence reads to genomic features. Bioinformatics.

[72] Davis S., Meltzer P.S. (2007). GEOquery: A bridge between the Gene Expression Omnibus (GEO) and BioConductor. Bioinformatics.

[73] Lohse M., Nunes-Nesi A., Kruger P., Nagel A., Hannemann J., Giorgi F.M., Childs L., Osorio S., Walther D., Selbig J. (2010). Robin: An intuitive wizard application for R-based expression microarray quality assessment and analysis. Plant Physiol..

[74] Hunt G.P., Grassi L., Henkin R., Smeraldi F., Spargo T.P., Kabiljo R., Koks S., Ibrahim Z., Dobson R.J.B., Al-Chalabi A. (2022). GEOexplorer: A webserver for gene expression analysis and visualisation. Nucleic Acids Res..

[75] Smyth G.K. (2004). Linear models and empirical bayes methods for assessing differential expression in microarray experiments. Stat. Appl. Genet. Mol. Biology..

[76] Kolde R., Laur S., Adler P., Vilo J. (2012). Robust rank aggregation for gene list integration and meta-analysis. Bioinformatics.

[77] Franceschini A., Szklarczyk D., Frankild S., Kuhn M., Simonovic M., Roth A., Lin J., Minguez P., Bork P., von Mering C. (2013). STRING v9.1: Protein-protein interaction networks, with increased coverage and integration. Nucleic Acids Res..

[78] Shannon P., Markiel A., Ozier O., Baliga N.S., Wang J.T., Ramage D., Amin N., Schwikowski B., Ideker T. (2003). Cytoscape: A software environment for integrated models of biomolecular interaction networks. Genome Res..

[79] Smoot M.E., Ono K., Ruscheinski J., Wang P.L., Ideker T. (2011). Cytoscape 2.8: New features for data integration and network visualization. Bioinformatics.

