# Supplementary material for: Transcriptional Dynamics of Receptor-Based Genes Reveal Immunity Hubs in Rice Response to Magnaporthe oryzae Infection
Source: Int J Mol Sci. 2025 May 12;26(10):4618. doi: 10.3390/ijms26104618 (PMC12111697; doi:10.3390/ijms26104618)
Supplement: Supplementary file 1 [file ijms-26-04618-s001.zip › Supplementary Figure 1. RNA data processing NCBI .pdf]

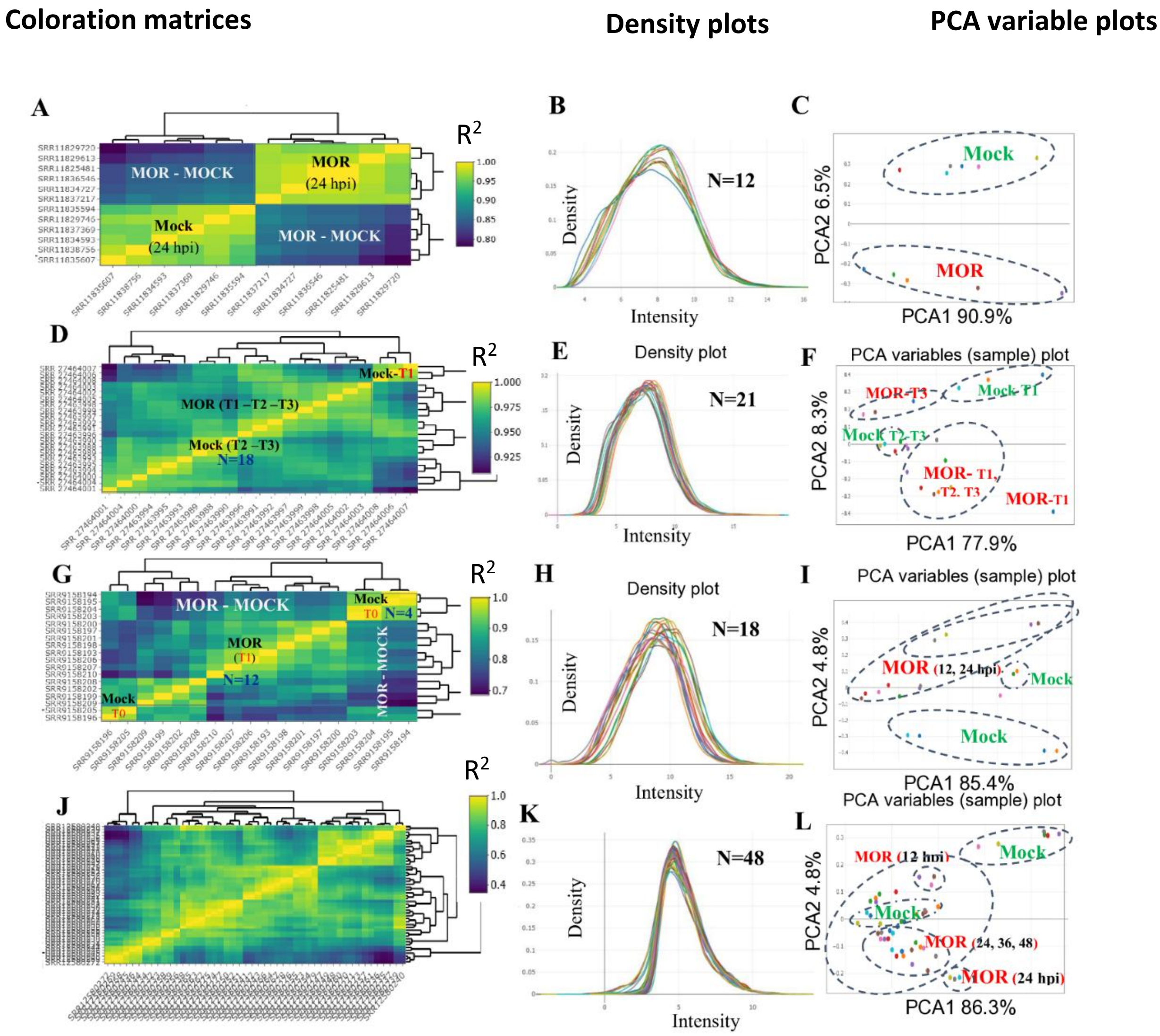

Figure S1. Overview and exploratory data analysis of RNA-seq samples dataset of rice transcriptome profile from the NCBI database. A, D, G, J: Pearson's correlation coefficients illustrate variation between groups. **B, E, H, K:** Density plots illustrating a nearly unimodal distribution. **C, F, I, L:** Principal component analysis (PCA) illustrates the top PCA components (PCA1 & PCA2). A-C: Bio-project PRJNA634330. D-F: Bio-project: PRJNA1062412, J-L: Bio-project: PRJNA545418. G-I: Bio-project: PRJNA661210, M-P:
