# Supplementary material for: Transcriptional Dynamics of Receptor-Based Genes Reveal Immunity Hubs in Rice Response to Magnaporthe oryzae Infection
Source: Int J Mol Sci. 2025 May 12;26(10):4618. doi: 10.3390/ijms26104618 (PMC12111697; doi:10.3390/ijms26104618)
Supplement: Supplementary file 1 [file ijms-26-04618-s001.zip › Supplementary Figure 2. Microarray pre-exporatory data.pdf]

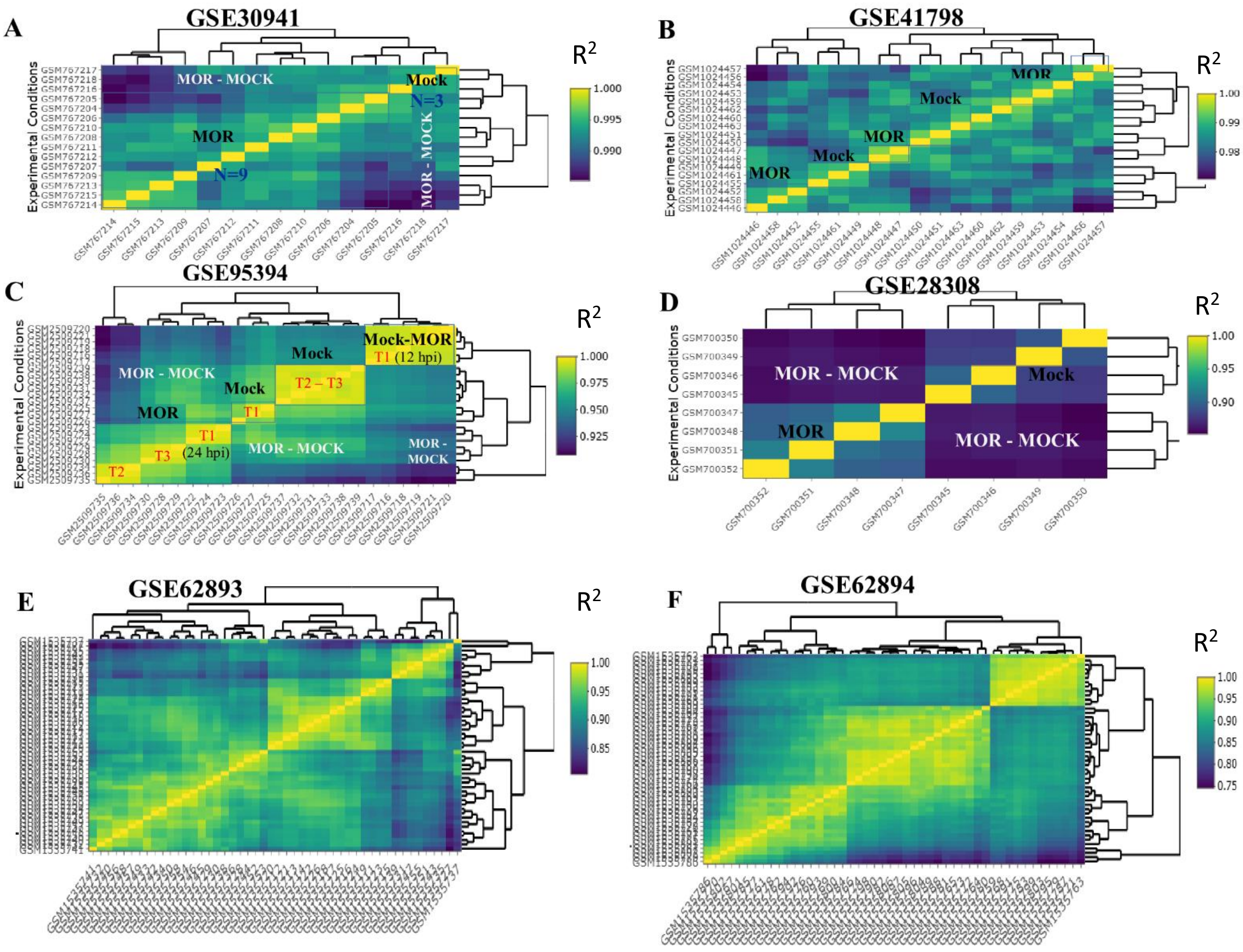

Figure S2. Overview and exploratory analysis of rice microarray sets infected by MOR infection. A, B, C, D, E: Correlation heatmap plot of Affymetrix GPL2025 microarray dataset. **G, H:** Correlation matrix plot of Affymetrix GPL6864 - microarray dataset. Distance matrix between samples based on Pearson's correlation coefficient. GPL2025 platform (Figs. A-D): GSE30941, GSE41798, GSE95394, GSE18361 and GSE18361. GPL6864 platform (Figs. E-F): GSE62893 and GSE62894.
