# Supplementary material for: Transcriptional Dynamics of Receptor-Based Genes Reveal Immunity Hubs in Rice Response to Magnaporthe oryzae Infection
Source: Int J Mol Sci. 2025 May 12;26(10):4618. doi: 10.3390/ijms26104618 (PMC12111697; doi:10.3390/ijms26104618)
Supplement: Supplementary file 1 [file ijms-26-04618-s001.zip › Supplementary Figure 3. pre-data microarray (GPL2025) .pdf]

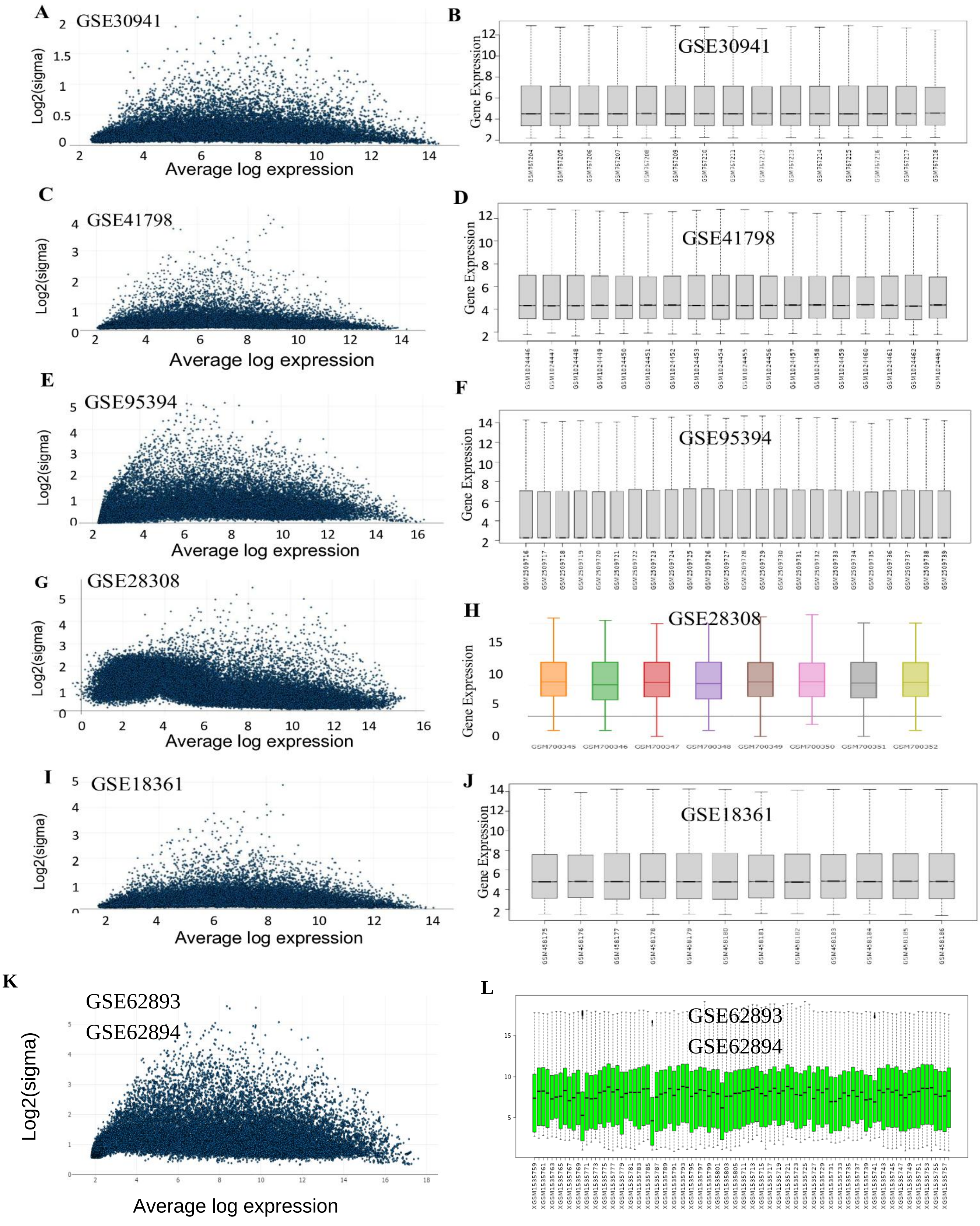

Figure S3: Overview and exploratory data analysis of microarray sets. A, C, E, G, I, K: Mean-variance illustrating the mean-variation relationship of the expression data. **B, D, F, H, J, L;** Whisker and box plot illustrating the median-centered values of the genes and disruption excretion values in the data set. **GPL2025 platform (A-J):** GSE30941, GSE41798, GSE95394, GSE18361 and GSE18361. **GPL6864 platform (K-L):** GSE62893 and GSE62894.
