# Supplementary material for: Transcriptional Dynamics of Receptor-Based Genes Reveal Immunity Hubs in Rice Response to Magnaporthe oryzae Infection
Source: Int J Mol Sci. 2025 May 12;26(10):4618. doi: 10.3390/ijms26104618 (PMC12111697; doi:10.3390/ijms26104618)
Supplement: Supplementary file 1 [file ijms-26-04618-s001.zip › Supplementary Figure 4. MAPK profile.pdf]

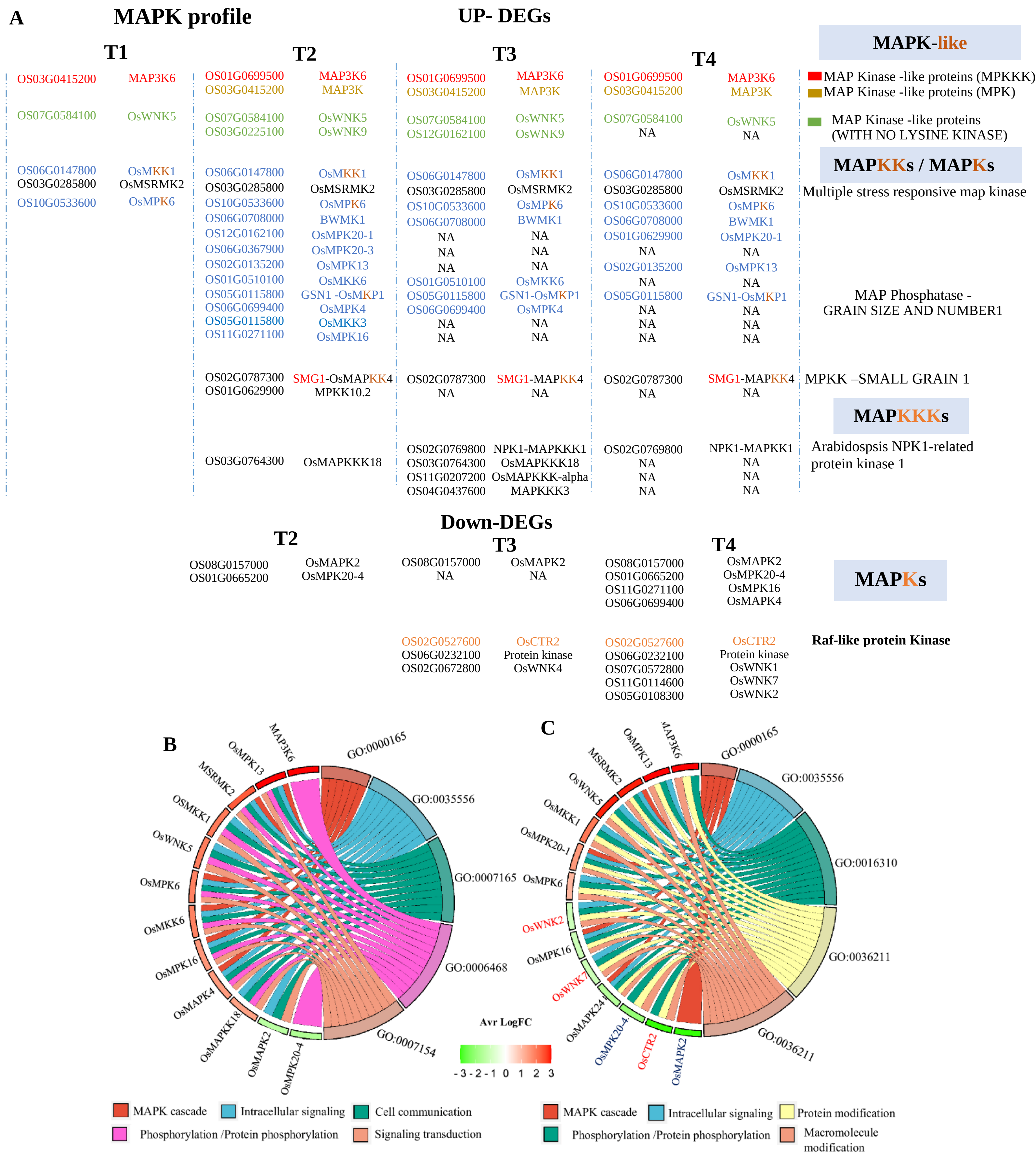

Figure S4. MAPK scaffolding profile of rice DEGs (**A**) responsive to MOR infection. **B, C:** Top enriched biological process (BP) gene ontology terms of MAPK genes. Two MAPK-like genes, including MAP3K6 and

OsWNK5 (WITH NO LYSINE KINASE), were commonly up-regulated in all the transcriptome time points. Two MAPK genes (*MPK6*, *MSRMK2*) and two MAPKK genes (*OsMKK1*, *SMG1-OsMAPKK4*) were commonly up-regulated across all transcriptomes. For the down DE genes, *OsMAPK2* was observed at T2-T4, while two MAP-like protein kinases, including Raf-like kinase (*OsCTR2*) and another kinase gene (*OS06g0232100*), were commonly identified at T3 and T4.
